# Crosstalk of RNA Adenosine Modification-Related Subtypes, Establishment of a Prognostic Model, and Immune Infiltration Characteristics in Ovarian Cancer

**DOI:** 10.3389/fimmu.2022.932876

**Published:** 2022-06-28

**Authors:** Xiaoge Ni, Can Chen, Guoliang Cui, Wei Ding, Jinhui Liu

**Affiliations:** ^1^Department of Obstetrics and Gynecology, Affiliated People’s Hospital of Jiangsu University, Zhenjiang, China; ^2^Department of Laboratory Medicine, The First Affiliated Hospital of Nanjing Medical University, Nanjing, China; ^3^Department of Gastroenterology, The Second Affiliated Hospital of Nanjing University of Chinese Medicine, Nanjing, China; ^4^Department of Nuclear Medicine, The First Affiliated Hospital of Nanjing Medical University, Nanjing, China; ^5^Department of Gynecology, The First Affiliated Hospital of Nanjing Medical University, Nanjing, China

**Keywords:** RNA modification “writers”, ovarian cancer, immune infiltration, RMW score, drug sensitivity

## Abstract

**Background:**

Four RNA adenosine modifications, including m6A, m1A, alternative polyadenylation, and adenosine-to-inosine RNA editing, have been identified as potentially valuable in influencing colorectal carcinogenesis, immune infiltration, and response to drug therapy. However, the regulatory mechanisms and clinical significance of these four RNA modifications in ovarian cancer (OC) remain unknown.

**Methods:**

We comprehensively described the transcriptional and genetic modifications of 26 RNA modification “writers” in OC and assessed the expression patterns. We identified two RNA modification subtypes using an unsupervised clustering approach. Subsequently, using differentially expressed genes (DEGs) in both subtypes, we calculated RNA modification “writer” scores (RMW scores) to characterize the RNA modifications of single OC patients. RMW score-related gene expression was investigated by qRT-PCR. We explored the correlation between RMW score and clinical features, immune infiltration, and drug sensitivity. We drew a nomogram to more intuitively and accurately describe the application value of the RMW score.

**Results:**

We found that molecular alterations in “writers” are strongly related to prognostic and immune-infiltrating features in OC patients. We identified two different clusters of RNA modifications. According to the immune infiltration characteristics in the two RNA modification isoforms, cluster A and cluster B can correspond to “hot” and “cold” tumors, respectively. With the median RMW score, we classified the patients into high- and low-score subgroups. A low RMW score was associated with good patient prognosis and lower immune infiltration. In addition, a low RMW score equated with a higher cancer stem cell index and a lower tumor mutation burden, which to some extent affected the sensitivity of patients to therapeutic drugs. Seven RMW score-related gene expressions were investigated by qRT-PCR in three OC cell lines. Compared to previously known models, our established RMW score has higher accuracy in predicting patient survival.

**Conclusion:**

A comprehensive analysis of four RNA modification patterns in OC reveals their potential value in OC prognosis, immune microenvironment, and drug sensitivity. These results could deepen our knowledge of RNA modification and yield fresh insights for new personalized therapeutic strategies.

## Introduction

Ovarian cancer (OC) is a malignant tumor that grows on the ovaries, 90%–95% of which are primary (1). Because the ovary is located in the pelvis, the onset is insidious, and there is no perfect early diagnosis and identification method. Once symptoms appear, it is often a late-stage disease (2). Of newly diagnosed OC patients, 70%~80% can achieve a certain curative effect, but because OC easily spreads and metastasizes in the abdominal cavity, most advanced OC patients will still face tumor recurrence (3). OC has the highest mortality rate among gynecological malignancies and has become the most threatening to women’s life and health among all gynecological tumors (4).

Like epigenetic DNA and histone modifications, RNA modifications have become important modulators of gene expression throughout eukaryotic development. So far, several kinds of RNA modifications have been recognized, including N6-methyladenosine (m6A) and N1-methyladenosine (m1A) (5). These modifications can be installed, removed, and decoded in a reversible manner *via* their specific cellular compositions and perform critical functions in multiple biotic processes (6). All RNA nucleotides, including adenine nucleotides, guanine nucleotides, cytosine nucleotides, and uracil nucleotides, are chemically modified (7). Among them, the modification of adenine nucleotide (A) is the most common, such as m1A, m6A, alterative polyadenylation (APA), and adenosine-to-inosine RNA editing (A-to-I). The RNA epigenetic modifications on these A bases are very different in catalytic principle and occurrence position (8–11) and generally do not compete to occur at the same A base position. However, some studies have elucidated the negative regulation of m6A modification on A-to-I editing and its mechanism (12). Considering the interaction between different RNA modifications and the fact that these modifications are mainly regulated by methyltransferases (writers) (13), we set out to study the regulatory network among the RNA “writers” with the above four modifications.

m6A is a methylation modification on the 6th nitrogen atom of adenine (14). m6A methylation is currently the most important chemical modification found in eukaryotic cells and plays an important role in various cellular processes, especially tumor development (15–17). During transcription m6A deposition occurs in nascent pre-mRNAs by methyltransferase complexes in the nucleus. Complexes include METTL3, METTL14, RBM15, WTAP, and KIAA1429, among others (18).

m1A is a methylation modification that occurs on the first nitrogen atom of adenine. A high abundance of m1A modifications is present on tRNA and rRNA (19, 20). Furthermore, m1A modifications also occur on mRNA (21). m1A plays a key role in regulating mRNA translation initiation and elongation, mRNA stability, and related developmental processes (22). The m1A “writers” that have been found so far mainly include TRMT10C, TRMT6, TRMT61A, and TRMT61B (21, 23).

APA is a widespread gene post-transcriptional regulation process in eukaryotes. Most APAs occur in the 3′UTR region (24). Through the selection of different polyadenylations in the 3′-UTR region, APA can affect important processes such as mRNA stability, translation efficiency, and cellular localization (25). The factors that regulate the formation of APA mainly include CFI, NUDT21, CPSF, PABPN1 family (CPSF1-4), and CTSF family (CSTF1-3) protein complexes (11, 24).

A-to-I type RNA editing is a fundamental biological phenomenon that is widespread in mammals and is considered a post-transcriptional modification mechanism capable of generating molecular diversity (26). It regulates protein translation by recoding, greatly enriching genetic information. A-to-I type RNA editing not only has an important impact on the regulation of gene expression but also is intimately linked to the pathogenesis of many diseases (27, 28). ADAR, ADARB1, and ADARB2 are the catalytic enzymes that exercise this important type of RNA modification (29).

The tumor immune microenvironment (TME) refers to the immediate ecological niche surrounding a tumor, consisting of various types of cells in the metabolic environment. TME contains a complex immune cellular environment that includes cells engaged in the innate immune response, such as natural killer (NK) cells and dendritic cells, and cells engaged in the adaptive immune response, such as T and B cells (30, 31). Some studies have classified tumors into “cold tumors” and “hot tumors” according to the presence or absence of tumor-infiltrating lymphocytes (TILs) in the TME. “Hot” tumors are tumors with infiltrating lymphocytes, whereas “cold” tumors are the opposite. In general, hot tumors are more immunogenic than “cold” tumors (32, 33). Recent findings suggest that RNA modifications are an essential epigenetic regime affecting tumor immune response and tumorigenesis (34). METTL3 deletion disrupts T-cell homeostasis and differentiation. METTL3-deficient T cells fail to perform homeostatic proliferation and maintain naïve (35). Four types of RNA modification “writers” have been shown to form a complex regulatory network in colorectal cancer to influence immune regulation and immunotherapy in the TME (36). However, whether this regulatory network plays an effect on OC TME is still unknown and needs to be further explored.

In this study, we assessed expression levels and genomic alterations in 26 “writers” in OC specimens from The Cancer Genome Atlas (TCGA) and Gene Expression Omnibus (GEO) databases, and we compared expression levels with normal ovarian samples from the GTEx database. By comprehensively evaluating the two RNA modification patterns of OC samples, we revealed that RNA modification modes are related to not only tumor immune infiltration, which can correspond to different immune typing, but also cell proliferation and oncogenic mechanisms. Patients were then categorized into distinct gene clusters according to the expression profiles of differentially expressed genes (DEGs) in two RNA modification clusters. Considering individual differences in RNA modification, we calculated the RNA modification score to accurately quantitate RNA modification patterns in a single OC patient and proved that this score can correctly predict patient outcomes, immune characteristics, and treatment efficacy.

## Materials and Methods

### Data Collection and Processing

The process of this research is illustrated in **Figure S1A**. Gene expression data and full clinical descriptions for OC were retrieved and obtained from GEO and TCGA databases. Somatic mutation and copy number variation (CNV) datasets were obtained from TCGA database. This study used three cohorts, TCGA-OV, GSE9891, and GSE26193, for subsequent analysis. Only tumor samples were retained for this study. Duplicate samples from the same patient were removed. Samples with no follow-up information and incomplete clinical information were also deleted. For TCGA-OV cohort, after converting the genes’ fragments per kilobase (FPKM) values to transcripts per kilobase (TPM), the “normalizeBetweenArrays” function of the R package “Limma” were applied to perform data normalization. For the GEO dataset, probe IDs were converted to gene symbols according to the platform annotation file. Normalized expression values were log-transformed and scaled before being used for model validation. The mean value of genes with multiple probes was used as their expression value (37). Normalization and removal of batch effects between TCGA-OV and two GEO datasets were performed using the “ComBat” algorithm from the “sva” package (38).

### Unsupervised Cluster Analysis of RNA Modification “Writers”

A total of 26 RNA modification “writers” were identified based on previous research (36). **Table S1** shows the details of these genes. According to these gene expression profiles, the “ConsensusClusterPlus” package was used to perform an unsupervised cluster analysis of the patients and divided the samples into two distinct subtypes. For the major parameters in the “ConsensusClusterPlus” function, the following was set: the max cluste number (maxK) = 9, proportion of items to sample (pItem) = 0.8, proportion of features to sample (pFeature) = 1, cluster algorithm (clusterAlg) = hc/hierarchical, and distance = spearman. The above process is repeated 1,000 times to ensure the consistency of the classification (39).

### Gene Set Variation Analysis

To explore the biological functions between different RNA modification patterns, based on the “c2.cp.kegg.v6.2.symbols.gmts” gene set in the MsigDB database, with two RNA modification isoforms as phenotypic features, the Gene Set Variation Analysis (“GSVA”) package was used to determine biological process differences between different RNA modification (40).

### Assessment of Immune Infiltration

The Single-Sample Gene Set Enrichment Analysis (ssGSEA) algorithm was applied to estimate the immune infiltration of each OC sample, and an enrichment score was used to indicate the degree of enrichment of each immune cell (41). The CIBERSORT algorithm assesses the composition and relative proportions of tumor-infiltrating immune cells in OC samples (42). CIBERSORT results are available online (https://gdc.cancer.gov/about-data/publications/panimmune) (43). ESTIMATE algorithm was used to compute immune and stromal scores between subgroups to deduce tumor purity (44).

### Correlation Between RNA Modifications and Other Biological Processes

Rosenberg et al. built a set of gene sets associated with biological processes, including epithelial–mesenchymal transition (EMT) markers, DNA damage repair, nucleotide excision repair, and CD8 T-effector signature (45–47). A correlation analysis of these biological pathways with RNA modification isoforms and RMW scores was performed to reveal the potential biological effects of RNA modifications.

### Identification of Differentially Expressed Genes Between RNA Modification Isoforms and Functional Annotation

Empirical Bayesian methods in the “limma” package were used for identifying DEGs of different RNA-modifying isoforms (48). A total of 1,641 DEGs were screened using adjusted p-value < 0.05 as criteria. “clusterProfiler” was used to perform Gene Ontology (GO) and Kyoto Encyclopedia of Genes and Genomes (KEGG) functional enrichment analysis to explore the potential biological functions of these DEGs (49).

### Construction of RNA Modification Gene Signature

All OC patients were equally randomized into training and test groups, and then RNA modification-related RMW scores were constructed using the training group. First, in the training set, a univariate Cox regression analysis of 1,641 DEGs identified 10 RNA modification “writers”-related genes significantly linked to prognosis (p < 0.001). The model fit was then minimized using the least absolute shrinkage and selection operator (LASSO) regression analysis (50). Finally, by obtaining the seven central DEGs and their correlation coefficients through a multivariate Cox regression model, an RNA modification gene signature, called the RMW score, was constructed. RMW score = Σ(Expi * coefi), where Coefi and Expi represent the correlation coefficient and expression of each gene, respectively. The sample was categorized into the high and low groups by median score. The “survminer” package was applied to perform survival analysis between the two groups and plot the receiver operating characteristic (ROC) curve to evaluate the model’s precision.

### RNA Extraction and qRT-PCR

TRIZOL reagent (Thermo Fisher Scientific, Waltham, MA, USA) was used to isolate total RNA from cell lines, and Revert Aid First Strand cDNA Synthesis kit (Thermo Fisher Scientific, USA) was used to synthesize cDNA. GAPDH was chosen as the internal reference. The relative expression of the target gene was estimated using the 2^−ΔΔCT^ method. The primer sequences are listed in **Table S2**.

### Cell Culture

The OC cell lines (SKOV3, HO8910, and OVCAR3) were purchased from China Center for Type Culture Collection (CCTCC) and CRC/PUMC (Cell Resource Center, IBMS, and CAMS/PUMC). The normal ovarian cell line (IOSE) was obtained from Shanghai Yaji Biotechnology Co., Ltd. All cells were maintained in Dulbecco’s modified Eagle medium (DMEM) (Gibco; Thermo Fisher Scientific, Inc.) containing 10% fetal bovine serum (FBS) (Gibco; Thermo Fisher Scientific, Inc.) in a humidified incubator at 37°C and 5% CO_2_.

### Creation and Validation of Nomogram

The “rms” package was used to integrate clinical characteristics and risk scores and draw a nomogram to visualize the relationship between variables in the prediction model (51). The calibration curve is used to verify the predictive ability of the prediction model. The closer the curve is to the diagonal, the better the prediction effect. Decision curve analysis (DCA) assessed the clinical application of the model by calculating the net benefit rate.

### Phenotypes of DNA and RNA Differentiation

Cancer stem cell scores, including mRNA expression-based RNAs and DNA methylation-based DNAs, were designed to gauge cancer stem cell association (52). Scores range from 0 to 1. The closer the score is to 1, the stronger the degree of stemness and the lower the degree of differentiation. Both RNA and DNA scores were obtained from the xena browser (https://xenabrowser.net/datapages/).

### Drug Sensitivity Prediction

The CellMiner database is based on the 60 types of cancer cells (NCI-60) listed by the National Cancer Institute’s Center for Cancer Research (NCI) (53). The NCI-60 cell line is the most widely used cancer cell sample population for anticancer drug testing. The CellMiner database was queried for 22,379 identified gene expression data and drug sensitivity data (IC50) for 20,503 analyzed compounds in NCI-60 cell lines to analyze the sensitivity between genes and drugs. Tumor Immune Dysfunction and Exclusion (TIDE) score is based on the analysis of T-cell dysfunction under a high level of cytotoxic T-cell infiltration and T-cell rejection characteristic genes in immunosuppression, which can effectively predict the effect of immune checkpoint inhibitor (ICI) therapy. The TIDE score is composed of two components: dysfunction score and exclusion score. The higher the TIDE score, the worse the efficacy of ICIs and the shorter the survival of patients (54). The Genomics of Drug Sensitivity in Cancer (GDSC) database contains information on molecular markers of drug sensitivity in cancer cells, which is important for discovering potential targets for tumor therapy. The GDSC database can be used to examine the sensitivity between RMW scores and cancer drugs (55).

### Statistical Analysis

Spearman’s and distance correlation analyses were performed to estimate correlation coefficients between the expression of RNA modification “writers” and immune infiltrating cells. Wilcoxon’s test was performed to analyze the variation between the two groups. Survival curves were drawn using the Kaplan–Meier (K-M) method, and a log-rank test was performed to determine the significance of the differences. ROC curve was performed to verify the validity of the model. p < 0.05 was considered statistically significant. All data were processed using R 4.0.1 software.

## Results

### Genetic and Transcriptional Alterations in RNA Modification “Writers” in Ovarian Cancer

**Figure 1:**
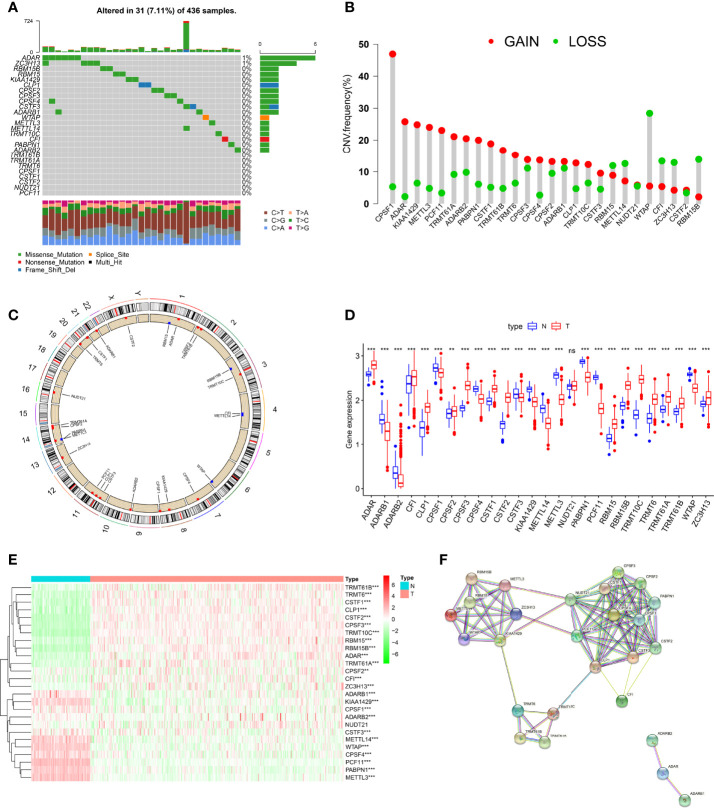
Genetic and transcriptional alterations of RNA modification “writers” in ovarian cancer (OC). **(A)** The mutation frequency of 26 RNA modification “writers” in 436 OC patients from The Cancer Genome Atlas (TCGA) cohort. **(B)** Frequencies of copy number variation (CNV) gain, loss, and non-CNV among RNA modification “writers.” **(C)** Locations of CNV alterations in RNA modification “writers” on 23 chromosomes. **(D)** Expression distributions of 26 RNA modification “writers” between normal and colorectal cancer (CRC) tissues. **(E)** Heatmap showing expression profiles of 26 RNA modification “writers” in OC and normal tissues. **(F)** The protein–protein interaction (PPI) network of RNA modification “writers”. Adjusted p-values were shown as ns, not significant; **p<0.01; ***p<0.001.

Altogether, 26 RNA modification “writers” were included in this study (**Table S1**) (15, 21, 24, 28, 36). First, we comprehensively analyzed the somatic mutation status of these “writers” in TCGA-OC cohort. Overall, 26 “writers” had low mutation rates in OC. Only 31 of 436 OC patients had RNA-modifying mutations (7.11%), and only ADAR and ZC3H13 mutations were present (**Figure 1A**). Next, we analyzed the CNVs and revealed that CNVs were ubiquitous in all “writers.” Among them, the vast majority of “writers”, such as CSAF and ADAR, showed copy number gain, while WTAP and others showed copy number loss (**Figure 1B**). **Figure 1C** shows the chromosomal locations of CNV variants in 26 “writers”.

To reveal whether genetic variation in these “writers” in OC interferes with gene expression, we compared these “writers” expressions in normal and OC tissues. Compared with normal tissues, the number of “writers” with increased, decreased expression was approximately equal in OC samples, and the difference in expression of these genes in both tissues was essentially statistically significant (**Figures 1D, E**). Previous studies have shown a coordination or inhibitory relationship between the four RNA modifications (12). These grooming functions are not completely independent, and the protein–protein interaction (PPI) network diagram shows the interrelationships that exist between these “writers” (**Figure 1F**). Subsequently, after further analysis, we found that there is a certain correlation between CNV changes and the expression of “writers.” CNV increases in genes such as CPSF1, KIAA1429, METTL3, and PCF11 were often accompanied by decreased expression levels, whereas ADAR, TRMT61A, and CSTF1, among others, showed the opposite. Likewise, “writers” with CNV loss also showed increased or decreased expression levels (**Figures 1B, D**). These results suggest that expression levels of RNA modification “writers” are affected by CNV, but CNV is not the only factor affecting gene expression (56); other factors, including multiple epigenetic modifications, also largely regulate gene expression (57).

The above analysis shows that the expression and genetic changes of RNA modification “writers” in normal and OC are highly heterogeneous, implying that RNA modification “writers” play a certain role in the occurrence and pathogenesis of OC.

### Determination of RNA Modification “Writers” Patterns in Ovarian Cancer

**Figure 2:**
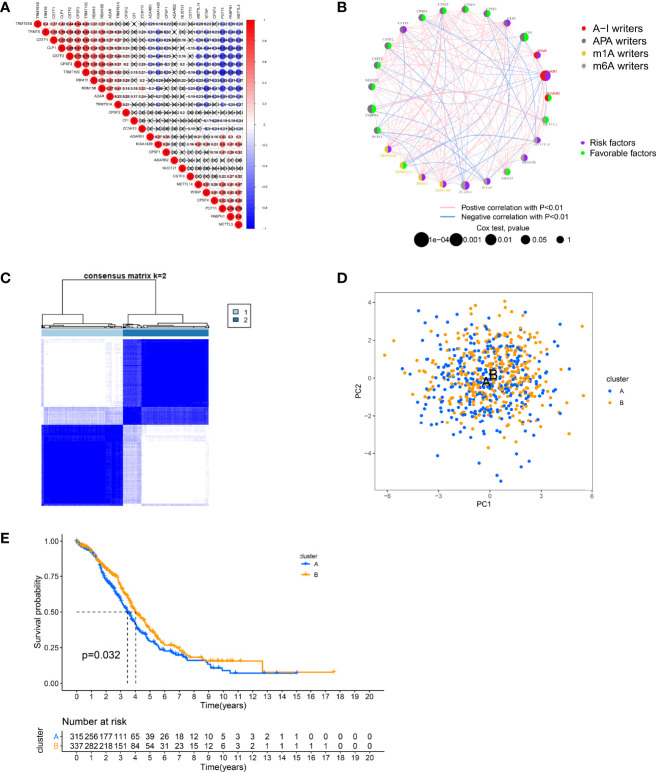
Patterns of RNA modification and clinical characteristics divided by consistent clustering. **(A)** Heatmap shows positive (red) and negative (blue) correlations among RNA modification “writers” in ovarian cancer (OC). **(B)** Interactions among RNA modification “writers” in OC. **(C)** Unsupervised clustering of RNA modification “writers” and Consensus matrix heatmaps for k = 2. **(D)** Principal component analysis (PCA) showing a remarkable difference in transcriptomes between different modification patterns. **(E)** Kaplan–Meier curves for the two RNA modification patterns of OC patients.

To better characterize RNA modifications in the development of OC, we integrated samples from TCGA-OC and GSE9891 cohorts and used them for further analysis. The Kaplan–Meier analysis revealed that 14 “writers” expression levels correlate with OC survival (p < 0.05, **Figure S1B**). Subsequently, we analyzed the relevance of expressions between “writers” and discovered a general agreement between positive and negative relationships (**Figure 2A**). The expression of “writers” was significantly correlated not only within the same category but also across different categories. Interestingly, the negative correlations between the expression of PCF11, PABPN1, METTL3, and other “writers” were relatively strong. The opposite is true for TRMT10C, CPSF3, and RBM15B. Likewise, the synthesis of “writers” interactions and prognostic value were demonstrated in the RNA modification network (**Figure 2B**). Therefore, the crosstalk among “writers” may largely influence RNA modification patterns in OC.

Subsequently, according to 26 “writers” mRNA expression profiles, we employed a consensus clustering algorithm to classify OC samples and divided the entire cohort into two clusters, including cluster A (n = 321) and cluster B (n = 340) (**Figures 2C**, **S2A, B**). Principal component analysis (PCA) showed that cluster A and cluster B could be well distinguished based on this classification (**Figure 2D**). The heatmap illustrates the possible relevance of the expression of RNA modification “writers” to some clinical traits (**Figure S2C**). Moreover, the K-M analysis revealed a more significant survival advantage for cluster B (p = 0.032, **Figure 2E**).

### Immune Signatures of Distinct RNA Modification Clusters

**Figure 3:**
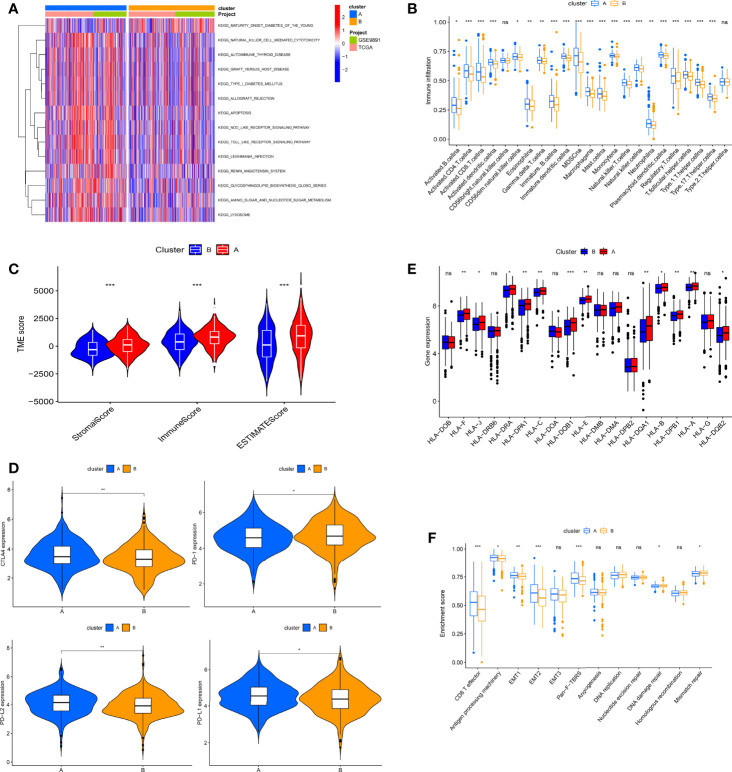
Biological characteristics and tumor immune microenvironment (TME) cell infiltration in two RNA modification patterns of ovarian cancer (OC). **(A)** Gene Set Variation Analysis (GSVA) analyzed the biological pathways between two modification patterns. **(B)** 23 TME cells’ infiltration abundance of two RNA modification patterns. **(C)** Correlations between two RNA modification patterns and TME score. **(D)** The RNA expression levels of HLA genes in samples from two patterns. **(E)** Expression levels of CTLA4, PD-1, PD-L2, and PD-L1 in two modification patterns. **(F)** Differences in interstitial activation pathways of two RNA modification patterns. Adjusted p-values were shown as ns, not significant; *p < 0.05; **p < 0.01; ***p < 0.001.

To gain insight into the potential biological meaning of two clusters, we conducted GSVA on two clusters. As can be seen from **Figure 3A**, some immune activation-related pathways are abundantly enriched in cluster A, including NK cell-mediated cytotoxicity and NOD-like and Toll-like receptor signaling pathways, indicating that these “writers” may be linked to immune activation. Therefore, we next explored the role of “writers” in OC TME. First, we performed ssGSEA in OC to assess immune infiltration in these clusters according to immune cell-specific marker gene expression levels. We found that cluster A was very rich in immune infiltration, with a significantly higher degree of infiltration than cluster B. Innate and adaptive immune cells including T and B cells, macrophages, and NK cells were significantly enriched in cluster A (**Figure 3B**). We then evaluated the correlation among the two RNA modification isoforms and 22 immune cell subpopulations with the CIBERSORT algorithm. Surprisingly, we found no difference between the two subtypes in most immune cell infiltrations (**Figure S2D**). This may be due to different algorithms. Furthermore, we employed the ESTIMATE algorithm to infer the proportions of immune cells and stromal cells in both subtypes and calculate tumor purity. The results showed that stromal cells and immune cells were significantly more abundant in cluster A, which also indicated that the tumor purity of cluster A was relatively low (**Figure 3C**). Based on these analyses, we found that the two RNA modification patterns have completely different immune infiltration characteristics. Among them, cluster A roughly corresponds to “hot” tumors, characterized by more activated immune cell infiltration and better response to immunotherapy, while cluster B corresponds to “cold” tumors, characterized by few infiltrating immune cells and a weak response to immunotherapy. However, cluster A with this immune signature did not have a matching survival advantage (**Figure 2E**).

To explore the expression characteristics of immune-related genes, we next analyzed the link between the immune checkpoint and HLA genes in both clusters. Immune checkpoint analysis revealed that, with the exception of PD-1, the remaining single genes, including PD-L1, CTLA-4, and PD-L2, were more highly expressed in cluster A (**Figure 3D**). Notably, all differentially expressed HLA genes were the highest in cluster A (**Figure 3E**). In addition, we further analyzed the correlation of known biological processes with the two isoforms in order to better characterize RNA modification patterns. The results showed that most biological processes were more prominent in cluster A, but pathways related to mismatch repair, including DNA replication, DNA damage repair, nucleotide excision repair, and mismatch repair, were significantly enriched in cluster B (**Figure 3F**).

### Construction of RNA Modification “Writer” Gene Clusters

**Figure 4:**
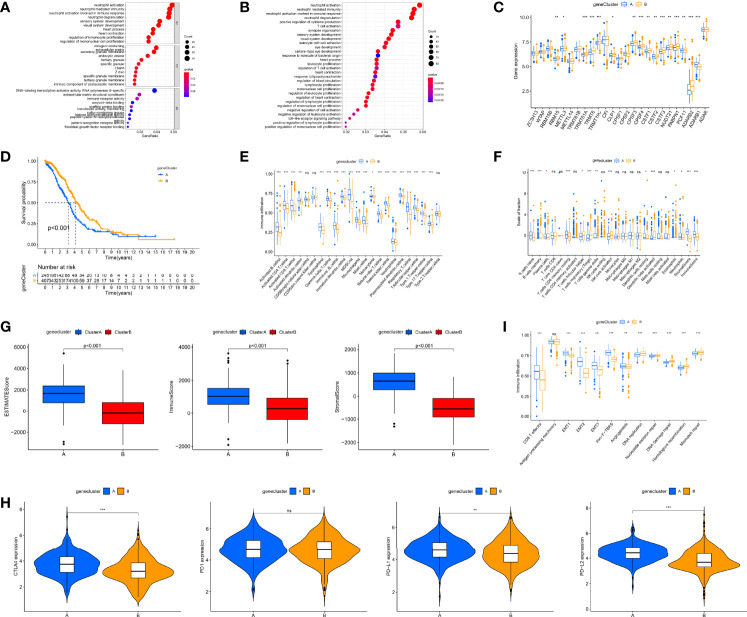
Identification of gene clusters based on differentially expressed genes (DEGs) and tumor immune microenvironment (TME) cell infiltration characteristics and transcriptome traits in distinct gene clusters. **(A)** Gene Ontology (GO) enrichment analyses of DEGs among two gene clusters. **(B)** Kyoto Encyclopedia of Genes and Genomes (KEGG) enrichment analyses of DEGs among two gene clusters. **(C)** Differences in the expression of 26 RNA modification “writers” among the two gene clusters. **(D)** Kaplan–Meier curves for the two gene clusters of ovarian cancer (OC) patients. **(E)** 23 TME cells’ infiltration abundance of two gene clusters. **(F)** The proportion of each immune cell in two gene clusters. **(G)** Correlations between two gene clusters and TME score. **(H)** Expression levels of CTLA4, PD-1, PD-L1, and PD-L2 in two gene clusters. **(I)** Differences in interstitial activation pathways of two gene clusters. Adjusted p-values were shown as ns, not significant; *p < 0.05; **p < 0.01; ***p < 0.001.

We used the “limma” package to screen out 1,641 DEGs (**Figure S3A**) and performed GO and KEGG functional analysis on DEGs. Gene enrichment analysis demonstrated that these DEGs were dramatically abundant in cell activation, proliferation, and immune-related pathways, including T-cell activation, lymphocyte proliferation, and neutrophil-mediated immunity (**Figures 4A, B**). This also indirectly indicates that RNA modification “writers” are essential in OC immune regulation. Subsequently, to determine the prognostic worth of these DEGs, we performed a univariate Cox analysis on these DEGs and screened out 10 genes associated with overall survival (OS) (p < 0.001, **Table S3**). Based on these 10 survival-related DEGs, we also used a consensus clustering algorithm to categorize OC simples into two gene clusters, namely, gene clusters A and B (**Figures S3B–D**). The expression of RNA modification “writers” differed between the two groups, with “writers” having relatively high expression in cluster B (**Figure 4C**). Consistent with the RNA modification cluster, the two gene clusters also each had different clinical and prognostic characteristics (**Figure S3E**), among which the K-M curve displayed a more pronounced survival advantage for patients in cluster B (p < 0.001, **Figure 4D**).

Subsequently, we further investigated immunological behavior in the two gene clusters. The ssGSEA results indicated that the vast majority of immune cells had higher levels of infiltration in cluster A (**Figure 4E**). The CIBERSORT algorithm results showed that gene cluster A was mainly infiltrated by adaptive immune cells (T and B cells), while gene cluster B was mainly infiltrated by innate immune cells, including NK cells and monocytes (**Figure 4F**). The results of tumor purity analysis showed that gene cluster A tumor tissues had a high content of immune and stromal cells, which also represented low tumor purity (p < 0.001, **Figure 4G**). Consistent with RNA modification typing, except for PD-1, the expression of other immune checkpoints in gene cluster A was also significantly higher (**Figure 4H**). In addition, classical biological pathways, including CD8 T effector, EMT1-3, Pan-F-TBRS, and Angiogenesis, were more prominent in gene cluster A, while pathways related to mismatch repair were significantly enriched in gene cluster B (**Figure 4I**). Again, based on these immune signatures, we believe that gene cluster A roughly corresponds to “hot” tumors, while gene cluster B corresponds to “cold” tumors.

### Construction and Validation of RMW Score

**Figure 5:**
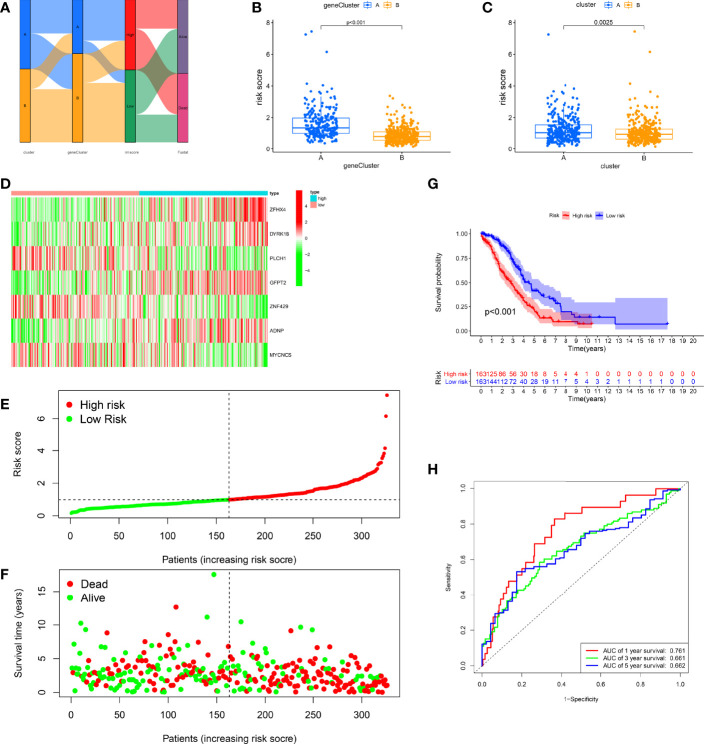
Construction of the RMW score in the training set. **(A)** Alluvial diagram of subtype distributions in groups with different RMW scores and survival outcomes. **(B)** Differences in RMW score between gene clusters. **(C)** Differences in RMW score between RNA modification patterns. **(D)** Heatmap shows the distribution of core genes in models between normal and ovarian cancer (OC) tissues. **(E)** Ranked dot showing the RMW score distribution and patient survival status. **(F)** Scatter plots showing the RMW score distribution and patient survival status. **(G)** Kaplan–Meier analysis of the overall survival (OS) between the two groups. **(H)** Receiver operating characteristic (ROC) curves to predict the sensitivity and specificity of 1-, 3-, and 5-year survival according to the RMW score.

Considering the complexity of RNA modification and individual differences, based on these DEGs, we created a scoring system to measure RNA modification patterns in single OC patients, called the RMW score. **Figure 5A** visually illustrates the distribution of sufferers across RNA modification clusters, genotypes, and RMW score groups. First, the expression profile data of the samples were merged with the survival information, and we selected 652 samples for subsequent analysis. These samples were averaged at random into a training group (n = 326) and a test group (n = 326) with the “caret” package. Based on previous genetic screening results, LASSO and multivariate Cox analyses were conducted on 10 prognostic-related DEGs in the training set to facilitate the selection of the best prognostic features. Nine OS-related DEGs were still screened by LASSO regression (**Figures S4A, B**). Subsequently, we applied multivariate Cox analysis to these 9 genes, and finally we obtained 7 genes for further analysis, including three low-risk genes (PLCH1, ZNF429, and MYCNOS) and four high-risk genes (ZFHX4, DYRK1B, GFPT2, and ADNP) (**Figure S4C**). Based on the correlation coefficient calculated by multivariate Cox regression, we established the calculation formula of RMW score: RMW score/risk score = (−0.1671 * PLCH1 expression) + (−0.6230 * ZNF429 expression) + (−0.2306*MYCNOS expression) + (0.1385 * ZFHX4 expression) + (0.5187 * DYRK1B expression) + (0.1590 * GFPT2 expression) + (1.0575 * ADNP expression).

Clearly, we observed significant differences in the distribution of RMW scores across different RNA modification clusters and gene clusters (**Figures 5B, C**). Among them, the RMW scores of cluster A and gene cluster A were relatively high, indicating that the high RMW score may be related to the immune infiltration and activation of patients. To estimate the clinically relevant nature of the RMW score, we categorized patients into high- and low-score (risk) groups by median RMW score. We observed significant expression variations in 7 genes between the two groups, with high-risk genes having higher expression in the high-score group, while low-risk genes showed the opposite (**Figure 5D**). **Figures 5E, F** show the distribution of RMW scores. It can be seen that the higher the RMW score, the shorter the patient’s survival time and the higher the mortality rate. The K-M survival curves highlight greater survival benefits for the high-score group (p < 0.001, **Figure 5G**). In addition, ROC curves illustrated the sensitivity and specificity of RMW score in predicting 1-, 3-, and 5-year survival, with area under the curve (AUC) values ​​of 0.761, 0.661, and 0.662, respectively (**Figure 5H**).

To further investigate the predictive behavior of the RMW score, we calculated the RMW score for the test and all sets (TCGA-OV+GSE9891) and the external validation group (GSE26193) according to the formula and categorized the patients into two score groups (**Figures S5**-**S9**). The heatmap illustrated the variation in RMW score-related gene expression among two groups (**Figures S5**-**9A**). **Figure S5**-**9B, C** show the change trend of RMW score and patient survival status in the two groups, respectively. Survival curves showed that the low-score group exhibited a significant survival benefit (p < 0.001; **Figures S5**-**9D**). ROC curves showed relatively high AUC values of RMW score in predicting 1-, 3-, and 5-year outcomes (**Figures3S5**-**9E**). These results suggest that the RMW score could mirror RNA modification patterns and forecast outcomes in OC.

We further validated the expression of 7 RMW score-related genes in OC cell HO8910, SKOV3, and OVCAR3 by qRT-PCR. As shown in **Figure S10**, the expressions of ADNP, PLCH1, ZFHX4, and ZNF429 were significantly lower in OC cell lines compared to those in IOSE cells. Meanwhile, DYRK1B and GFPT2 expressions were significantly upregulated in OC cell lines. However, there was no significantly different in MYCNOS expression in OC cell lines.

### Clinical Correlation Analysis and Stratified Analysis of RMW Score

We discussed the relevance of RMW scores to various clinical features, including survival status, age, and stage. We noticed that a greater proportion of patients over 60 years of age, with advanced disease and death, were in the high group (**Figures S11A–C**), and these patients had higher RMW scores (**Figures S11D–F**). In addition, stratified analysis assessed whether the RMW score could predict survival in different clinical subgroups, including age (≤60 and >60 years), grade (stages 1–2 and 3–4), stage (stages I–II and stages III–IV), BRCA1 (mutant and wild type), and chemotherapy (accepted and non-accepted). **Figures S11G–N** show that the low RMW score group has a more favorable prognostic outlook among patients stratified by various clinical traits. These results suggest that the RMW score can be employed to estimate several clinical characteristics, including age, stage, grade, and survival status.

### RMW Score Is Associated With Tumor Immune Microenvironment Immune Infiltration

**Figure 6:**
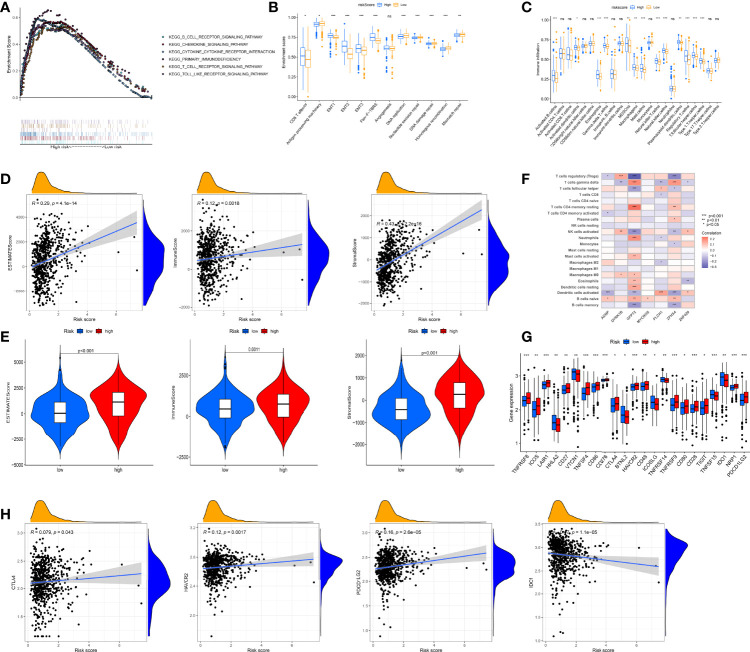
Evaluation of the tumor immune microenvironment (TME) and checkpoints between the two groups. **(A)** Enrichment plots showing B-cell receptor signaling pathway, chemokine signaling pathway, cytokine–cytokine receptor interaction, T-cell receptor signaling pathway, primary immunodeficiency, and Toll-like receptor signaling pathway were enriched in the high RMW score subgroup. **(B)** Differences in interstitial activation pathways of two groups. **(C)** 23 TME cells’ infiltration abundance of two RMW score subgroups. **(D, E)** Correlations between two groups and TME score. **(F)** Correlations between the abundance of immune cells and seven genes in model. **(G)** Expression of 23 immune-related genes in two groups. **(H)** Expression levels of CTLA4, HAVCR2, PDCD1LG2, and IDO1 in two groups. Adjusted p-values were shown as ns, not significant; *p < 0.05; **p < 0.01; ***p < 0.001.

First, we performed GSEA to identify the underlying biological properties of different scoring groups. As expected, significant enrichment of immune pathways, including T- and B-cell receptor pathways, chemokine signaling pathway, and cytokine–cytokine receptor interaction, was detected in high-risk patients compared with low-score patients (**Figure 6A**). Further studies showed that pan-F-TBRS and TME stroma were markedly activated in the high group, which, to some extent, mediated tumor immune tolerance (**Figure 6B**). Subsequently, we applied ssGSEA to assess immune infiltration in both groups. As depicted in **Figure 6C**, the level of immune cell infiltration was generally higher in the high group. To better illustrate the effect of the RMW score on TME immune infiltration, we also tested the correlation between different immune cells and the RMW score (**Figure S12A**). The CIBERSORT algorithm revealed that the RMW score was negatively linked to regulatory T cells, memory B cells, activated dendritic cells, and activated NK cells, while naive B cells, eosinophil, M0 macrophages, activated mast cells, plasma cells, and γδ T cells were positively correlated (**Figure S12B**). **Figure S12C** indicates the ratio of each immune cell in two groups. ESTIMATE results confirmed a positive relationship between RMW scores and both immune and stromal scores, indicating lower tumor purity in the high group (**Figures 6D, E**). Moreover, we assessed the relationship between seven RMW score-related genes and immune cell abundance and observed that the majority of immune cells were markedly linked to seven genes (**Figure 6F**).

Furthermore, we surveyed the connection between immune checkpoints and our risk model. **Table S4** lists 47 immune checkpoint-related genes. **Figure 6G** depicts 23 immune checkpoints with differential expression in both groups, including HAVCR2, IDO1, PDCD1LG2, and CTLA4, and most immune checkpoints were overexpressed in the high group. To better illustrate the characteristics between immune checkpoints and RMW scores, we also tested the correlation between some drug target genes and RMW scores (**Figures 6H**, **S12D**). In addition to IDO1, three other genes, including PDCD1LG2, HAVCR2, and CTLA4, were positively correlated with the RMW score.

### Correlation Between RMW Score and Tumor Stem Cells and Tumor Mutation Burden

Recently, studies have found that cancer stem cells interact with immune cells in the TME and can promote the progression of various cancers (58, 59). We analyzed the regulatory role of the RMW score in OC stem cells by analyzing mRNA expression (RNAs) and DNA methylation patterns (DNAs). It is evident from **Figures S13A, B** that the RMW score was clearly negatively dependent on both RNAs and DNAs, although these correlations were not statistically significant. These results suggest that a high RMW score is associated with reduced tumor cell stemness.

Growing evidence suggests that the higher the tumor mutation burden (TMB), the greater the number of neoantigens in the tumor, and the better the patient’s susceptibility to immunotherapy (60). Therefore, we next comprehensively evaluated the distribution of TMB in the two groups. **Figure S13C** highlights the higher TMB for the high group (p = 0.0072). Subsequently, the somatic mutation distribution results revealed a high mutation frequency in both groups, with the highest mutation frequency being in TP53, which can be as high as 88% and 80%. High score patients had a markedly higher frequency of mutations (**Figures S13D,E**).

### RMW Score Can Predict Drug Sensitivity in Ovarian Cancer Patients

**Figure 7:**
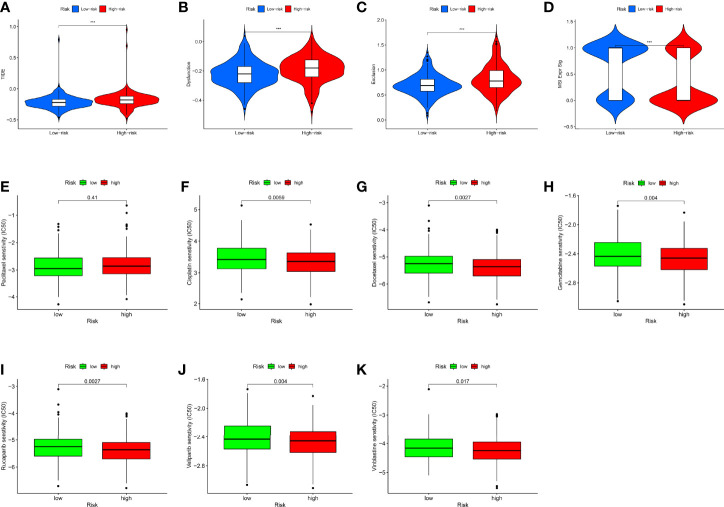
The role of RMW score in ovarian cancer (OC) treatment drug sensitivity. **(A–C)** The correlation between the RMW score and Tumor Immune Dysfunction and Exclusion (TIDE) Score. **(D)** Relationships between RMW score and microsatellite instability (MSI). **(E–K)** Relationships between RMW score and chemotherapeutic sensitivity. Adjusted p-values were shown as ***p < 0.001.

To explore whether the RMW score can predict the immunotherapy response to ICIs, we used the TIDE score to model the two main mechanisms of tumor immune evasion and provide predictive outcomes for immunotherapy. We found that TIDE scores in the high-score group were much higher, suggesting that the low RMW score patients were more likely to respond to immunotherapy (**Figures 7A–C**). Microsatellite instability (MSI) refers to the difference in the number of repeat units of the same microsatellite locus between different individuals or between normal tissues and some abnormal tissues of the same individual. Growing evidence indicates that patients with higher MSI are better able to respond to and gain from immunotherapy (61). From **Figure 7D**, we can see that the MSI was higher in the low RMW score group. This also proves that low-score patients with high MSI can benefit from immunotherapy.

We next selected chemotherapeutic agents available for OC to assess the sensitivity of the two groups to these agents. Interestingly, we found that with the exception of paclitaxel, the IC50 values ​​of the rest of the drugs, including rucaparib, veliparib, vinblastine, cisplatin, docetaxel, and gemcitabine, were significantly elevated in the low RMW score group (**Figures 7E–K**). **Figure S14** shows the correlation between RMW score-related genes and different drugs. These results suggested that RNA modification “writers” are associated with drug sensitivity, including immunotherapy and chemotherapeutics, where a lower RMW score suggests better treatment outcomes for patients.

### Plot a Nomogram to Predict Survival

**Figure 8:**
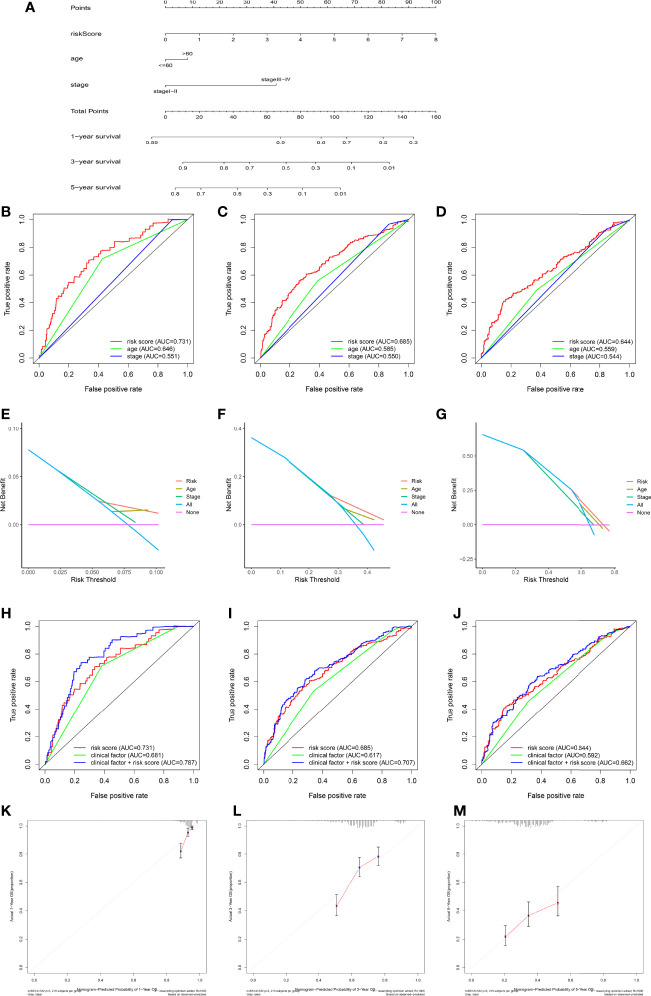
Construction and validation of nomograms. **(A)** Nomogram for predicting the 1-, 3-, and 5-year overall survival (OS) of ovarian cancer (OC) patients in the training set. **(B–D)** Receiver operating characteristic (ROC) curve showing the area under the curve (AUC) values of risk, nomogram, age, and stage in predicting survival in OC patients. **(E–G)** Decision curve analysis of the risk, age, and stage at 1-, 3-, and 5-year OS. The x-axis shows the threshold probability, and the y-axis measures the net benefit. **(H–J)** Receiver operating characteristic (ROC) curve showing the AUC values of risk score, clinical factors, and both sides in predicting survival in OC patients. **(K–M)** Calibration curves of the nomogram for predicting 1-, 3-, and 5-year OS.

Considering the inconvenient clinical application of RMW scores in predicting the prognosis of OC patients, we combined RMW scores with independent prognostic clinical traits, including age and stage, to draw a nomogram to more intuitively demonstrate the validity of these factors, especially RMW scores, in predicting 1-, 3-, and 5-year survival (**Figure 8A**). In addition, the conjoint univariate and multivariate analyses confirmed that the signature of the RMW score was an independent prognostic factor in five sets (**Table 1**). Considering the effect of sample number on the results, we used the samples from the ALL sets (TCGA-OV+GSE9891) to plot the nomogram and carry out the next step of the analysis. ROC results showed that the accuracy of OS at 1 year was higher than that at 3 and 5 years (**Figures 8B–D**). DCA showed that the net benefit of our RMW score-based prognostic model was high compared to clinical factors if the patient or physician threshold probability was greater than 50%, particularly when predicting 1-year survival. Within this range, the RMW score outperformed the predictions of individual predictors (**Figures 8E–G**). Subsequently, we plotted ROC curves to analyze the accuracy of combining the three predictors for a common prediction. Clearly, combining clinical factors with the RMW score predicted larger AUC values ​​with higher accuracy (**Figures 8H–J**). The calibration curves showed that the proposed nomogram was the best at predicting 1-year survival, followed by 3 years, compared to the ideal model (**Figures 8K–M**).

**Table 1:** Univariate and multivariate Cox regression analysis of RMW score and clinical characteristics in five cohorts.

	Univariate analysis				Multivariate analysis			
**Training set**								
Parameters	HR	HR.95L	HR.95H	pvalue	HR	HR.95L	HR.95H	pvalue
age	1.5809	1.1791	2.1196	0.0022	1.4109	1.0464	1.9022	0.0240
grade	1.1540	0.8078	1.6484	0.4312				
stage	4.3236	1.7715	10.5524	0.0013	3.6604	1.4979	8.9453	0.0044
RMW score	1.7699	1.5458	2.0264	0.0000	1.6933	1.4729	1.9466	0.0000
**Testing set**								
Parameters	HR	HR.95L	HR.95H	pvalue	HR	HR.95L	HR.95H	pvalue
age	1.3427	0.9763	1.8467	0.0699				
grade	1.3518	0.8890	2.0556	0.1586				
stage	3.9164	1.2448	12.3219	0.0196	3.5110	1.1138	11.0670	0.0320
RMW score	1.4056	1.2321	1.6035	0.0000	1.3829	1.2102	1.5802	0.0000
**TCGA set**								
Parameters	HR	HR.95L	HR.95H	pvalue	HR	HR.95L	HR.95H	pvalue
age	1.4054	1.0784	1.8317	0.0118	1.2402	0.9479	1.6225	0.1164
grade	1.1815	0.7885	1.7705	0.4189				
stage	2.4177	0.9945	5.8771	0.0514				
RMW score	1.8450	1.5814	2.1527	0.0000	1.8076	1.5451	2.1146	0.0000
**GSE9891 set**								
Parameters	HR	HR.95L	HR.95H	pvalue	HR	HR.95L	HR.95H	pvalue
age	1.5628	1.0743	2.2734	0.0196	1.3806	0.9383	2.0314	0.1017
grade	1.3024	0.8817	1.9236	0.1843				
stage	7.0056	2.2210	22.0977	0.0009	6.0557	1.9127	19.1730	0.0022
RMW score	1.4180	1.2441	1.6162	0.0000	1.3218	1.1481	1.5218	0.0001
**ALL set**								
Parameters	HR	HR.95L	HR.95H	pvalue	HR	HR.95L	HR.95H	pvalue
age	1.4563	1.1740	1.8066	0.0006	1.2840	1.0293	1.6019	0.0267
grade	1.2264	0.9353	1.6080	0.1399				
stage	4.0521	2.0069	8.1812	0.0001	3.5366	1.7494	7.1498	0.0004
RMW score	1.5346	1.4041	1.6773	0.0000	1.4718	1.3412	1.6150	0.0000

### RNA Modification “Writers” Models as Ovarian Cancer Novel Predictors

**Figure 9:**
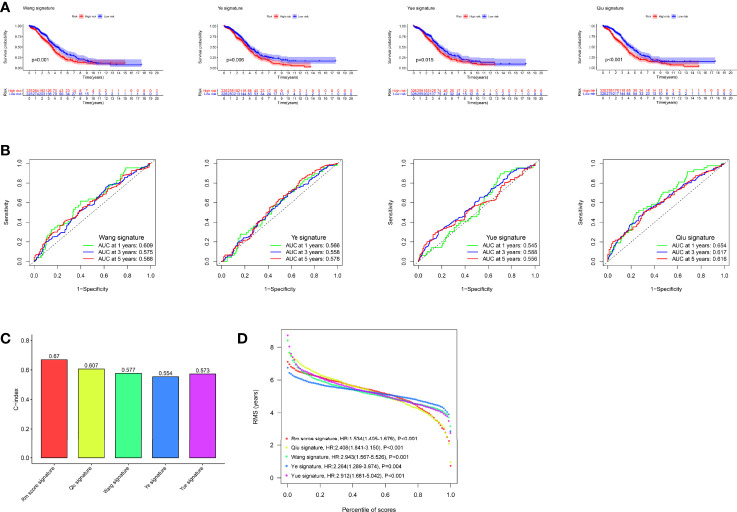
Comparison of our risk model with other established models. **(A)** Kaplan–Meier (K-M) curves of four other published gene signatures. **(B)** Receiver operating characteristic (ROC) curves of four other published gene signatures. **(C)** Concordance index (C-index) of the five prognostic risk models. **(D)** Restricted mean survival (RMS) time curve of all five prognostic risk models.

To compare the predictive performance of our RNA modification “writers” signature with other models, we selected four risk models, including 5-gene (62), 7-gene (63), 8-gene (64), and 11-gene (65) features. To make them comparable, according to the expression of corresponding genes in these four models, we also applied multivariate Cox regression analysis to calculate the risk value and prognostic evaluation for each dataset. Samples were divided into high and low groups according to the median value. Survival curves indicated that the high-risk group had a greatly worse prognosis in the four models (**Figure 9A**). ROC curves illustrated that the AUC values ​​were lower in all four models (**Figure 9B**). Therefore, we believe that they are inferior in predicting prognosis compared with our model. The restricted mean survival (RMS) package was employed to calculate the C-index for all prognostic features. Clearly, our model has the highest C-index at 0.67 (**Figure 9C**). With the use of RMS time, the predictive effect of gene signatures at different time points can be evaluated. Therefore, our genetic signatures perform best over time periods of about 5 years. This indicated that our model was the best predictor of 5-year survival in patients as compared with other models (**Figure 9D**).

## Discussion

To date, most studies have pointed to RNA modification as a key mechanism in the epigenetic regulation of immune responses and tumorigenesis. The dysregulation of m6A, m1A, APA, and A-to-I, four common RNA adenosine modifications mediated by “writers” enzymes, has been implicated in the pathogenesis of human diseases. However, most studies have focused on a single type of RNA modification “writers”. Thus, corporate effects and TME infiltration traits mediated by the combined action of multiple “writers” in OC have not been fully elucidated. In this study, we reveal the overall alterations and interactions of RNA modification “writers” at transcriptional and genetic levels in OC. Based on the expression levels of the 26 “writers”, we identified two distinct RNA modification clusters. Compared with cluster A patients, cluster B patients had milder clinicopathological features and a better prognostic outlook. There were significant differences in immune cell abundance in the TME between the two RNA modification subtypes. Among them, cluster A was characterized by abundant immune infiltration and significant immune activation, which contained a large number of T and B lymphocytes and was associated with immune-activated pathways, such as T- and B-cell receptor signaling pathways and NOD-like and Toll-like receptor signaling pathways. Moreover, transcriptome differences between RNA modification isoforms were markedly associated with cell proliferation and immune-related biological pathways. We identified two sets of gene clusters according to DEGs between two RNA-modification clusters. We believe that RNA modification “writers” may act as a major factor influencing the clinical outcome of OC and immune infiltration of the TME. We select “writers” that can robustly and effectively predict OC survival to calculate the RMW score. We demonstrate the RMW score’s predictive value and explore the expression of RMW score-related genes in OC tissues. RNA modification patterns characterized by high immune infiltration and immune activation showed higher RMW scores. Patients in the two groups exhibited significantly different clinicopathological features, prognosis, mutations, TME, immune checkpoints, CSC index, and drug sensitivity. Finally, by integrating age, stage, and RMW score, we built a nomogram to more intuitively display the performance of these factors and improve the applicability of the RMW score. This prognostic model might be employed for the prognostic stratification of OC patients, which helps to better understand the molecular mechanism of RNA methylation in OC and provides new ideas for personalized treatment.

Recently, immunotherapy has gradually achieved some advances in gynecological cancer. However, OC does not respond well to many immunotherapy drugs, and the immune characteristics of OC itself limit the response to immunotherapy and disease progression to a large extent (66). Previous studies have shown that the TME changes during malignant progression, mainly in non-malignant cells, cytokine networks, and the extracellular matrix. The heterogeneity of the TME may be one of the main reasons for the unsuccessful immunotherapy of OC (67). In this study, RNA modification patterns characterized by high immune infiltration and immune activation were associated with higher RMW scores. We found considerable variations in TME characteristics and relative abundance of immune cells between the two patterns, genotypes and different RMW score subgroups. This highlights an essential contribution of RNA modification “writers” in regulating the OC TME and progression.

Studies have proved that the presence of TILs is directly associated with a higher prognosis in OC patients (68). Among them, CD8+ T cells infiltrate tumors as a symbol of immune recognition and destroy tumor cells by secreting granzyme B, TNF, and IFNγ, indicating better survival in OC patients (69). B cells can also inhibit OC migration and metastasis through antitumor immunity to a certain extent, and some B cells differentiate into plasma cells to produce tumor-specific antibodies (70). Myeloid-derived suppressor cells (MDSCs) are a subset of immunoregulatory immature myeloid cells that proliferate throughout cancer progression and perform immunosuppressive functions by modulating the escape of antitumor T-cell immunity (71). In OC, MDSCs enhance cancer cell stemness and accelerate metastasis and tumor formation by triggering microRNA expression and inhibiting the co-suppressor gene C-terminal binding protein-2 (72). The high expression of immunosuppressive molecules, including vascular endothelial growth factor and interleukin 10 in OC TME, causes abnormal dendritic cell function, which not only fails to activate cytotoxic T lymphocytes but also induces regulatory differentiation of effector T lymphocytes, further suppressing the immune response of effector T lymphocytes to tumors (73). In addition, the extracellular matrix protein transforming growth factor beta (TGFBI), an essential component of the OC TME, is significantly upregulated in ovarian lesions and serous OC, which may contribute to immunosuppression and disease progression (74). We observed that subtype A had more immune cell infiltration, including antitumor cells such as T and B and tumor-promoting cells such as MDSCs and Treg. Likewise, we observed a similar phenomenon in the high RMW score group. However, subtypes with such high immune infiltration did not show a matching survival advantage. This indicates that in the immune microenvironment of OC, cells such as Treg and MDSCs may be primarily involved in suppressing the anticancer immune response. This is consistent with our finding of low immune infiltration and favorable prognosis in patients with subtype B and high RMW scores. T-cell exhaustion is characterized by loss of T-cell effector function, increased and persistent inhibitory receptor (IRS) expression, altered epigenetic and transcriptional profiles, and altered metabolic patterns (75). T-cell exhaustion is one of the major factors in immune dysfunction in cancer patients, including OC (76). Therefore, we speculate that the increased infiltration of T cells in the poor-prognosis cohort may be dominated by exhausted T cells, which indirectly suggests why high T-cell infiltration in the tumor worsens the prognosis. Exhausted T cells may have new targets for OC immunotherapy and represent a potent weapon to fight tumors.

With the deepening of tumor immunology and molecular biology research, immunotherapy and targeted therapy have become new directions of clinical research. Our findings suggest that RNA modification patterns may be considered appropriate “predictors” for assessing clinical outcomes of chemotherapy or targeted therapy. The correlation between RMW score and cancer stem cells can be used to predict patient responsiveness to immunotherapy. By identifying RNA modification signatures of individual tumors, our findings provide new possibilities for improving OC therapy and enabling personalized cancer treatment outcomes. However, the article still has shortcomings. First, all conclusions are derived from processing and retrospective analysis of data from public databases, lacking clinical data and experimental studies to validate the results. Furthermore, due to data limitations, our analysis lacked a large-scale clinical cohort to validate the correlation between RNA modifications and tumor immune infiltration and the prognostic value of the RMW score in OC. A large number of prospective clinical analyses are required for further validation in the future.

## Conclusion

Our comprehensive analysis of the four types of RNA modification “writers” uncovered broad regulatory mechanisms that influence TME infiltration characteristics and prognosis and identified their therapeutic utility in targeted therapy and immunotherapy. These findings emphasize the important clinical relevance of RNA modification “writers” and point to novel approaches for directing personalized treatment strategies for OC patients.

## Data Availability Statement

The datasets presented in this study can be found in online repositories. The names of the repository/repositories and accession number(s) can be found in the article/supplementary material.

## Author Contributions

JL and XN conceived the study and participated in the study design, performance, and manuscript writing. CC, GC, and WD conducted the bioinformatics analysis. All authors read and approved the final manuscript.

## Conflict of Interest

The authors declare that the research was conducted in the absence of any commercial or financial relationships that could be construed as a potential conflict of interest.

## Publisher’s Note

All claims expressed in this article are solely those of the authors and do not necessarily represent those of their affiliated organizations, or those of the publisher, the editors and the reviewers. Any product that may be evaluated in this article, or claim that may be made by its manufacturer, is not guaranteed or endorsed by the publisher.

## Supplementary Material

Supplementary Figure 1 Overview of study design and prognostic characteristics of RNA modification writers. **(A)** Overview of this work. **(B)** Kaplan-Meier survival analysis of different RNA modification “writers” in OC.

Supplementary Figure 2 Clinical and immune infiltration characteristics in two modification patterns. **(A)** Cumulative distribution function curves for unsupervised clustering of 26 RNA modification “writers”, k = 2-9. **(B)** Relative change in area under the CDF curve for unsupervised clustering of 26 RNA modification “writers”, k = 2-9. C Heatmap showing differences in clinicopathologic features and expression levels of RNA modification “writers” between the two modification patterns. **(D)** The proportion of each immune cell in two modification patterns.

Supplementary Figure 3 Clinical and immune infiltration characteristics in two gene clusters. **(A)**, 1641 RNA modification patterns-related DEGs shown in venn diagram. **(B)** Heat map of the consensus matrix for the OC sample at k = 2. C Cumulative distribution function curves for unsupervised clustering of DEGs, k = 2-9. **(D)** Relative change in area under the CDF curve for unsupervised clustering of DEGs, k = 2-9.**(E)** Heatmap showing differences in clinicopathologic features and expression levels of RNA modification “writers” between gene clusters.

Supplementary Figure 4 Identifying representative candidate prognostic genes. **(A-B)** The LASSO regression analysis and partial likelihood deviance on the prognostic genes. **(C)** Forest plot of multivariate cox regression analysis for prognostic genes.

Supplementary Figure 5 Validation of RMW score in testing set. **(A)** Heatmap shows the distribution of core genes in models between normal and OC tissues. **(B)** Ranked dot showing the RMW score distribution and patient survival status. **(C)** Scatter plots showing the RMW score distribution and patient survival status. **(D)** Kaplan–Meier analysis of the OS between the two groups. **(E)** ROC curves to predict the sensitivity and specificity of 1-, 3-, and 5-year survival according to the RMW score.

Supplementary Figure 6 Validation of RMW score in TCGA-OC set. **(A)** Heatmap shows the distribution of core genes in models between normal and OC tissues. **(B)** Ranked dot showing the RMW score distribution and patient survival status. **(C)** Scatter plots showing the RMW score distribution and patient survival status. **(D)** Kaplan–Meier analysis of the OS between the two groups. **(E)** ROC curves to predict the sensitivity and specificity of 1-, 3-, and 5-year survival according to the RMW score.

Supplementary Figure 7 Validation of RMW score in GSE9891 set. **(A)** Heatmap shows the distribution of core genes in models between normal and OC tissues. **(B)** Ranked dot showing the RMW score distribution and patient survival status. **(C)** Scatter plots showing the RMW score distribution and patient survival status. **(D)** Kaplan–Meier analysis of the OS between the two groups. **(E)** ROC curves to predict the sensitivity and specificity of 1-, 3-, and 5-year survival according to the RMW score.

Supplementary Figure 8 Validation of RMW score in whole set. **(A)** Heatmap shows the distribution of core genes in models between normal and OC tissues. **(B)** Ranked dot showing the RMW score distribution and patient survival status. **(C)** Scatter plots showing the RMW score distribution and patient survival status. **(D)** Kaplan–Meier analysis of the OS between the two groups. **(E)** ROC curves to predict the sensitivity and specificity of 1-, 3-, and 5-year survival according to the RMW score.

Supplementary Figure 9 Validation of RMW score in GSE26193 set. **(A)** Heatmap shows the distribution of core genes in models between normal and OC tissues. **(B)** Ranked dot showing the RMW score distribution and patient survival status. **(C)** Scatter plots showing the RMW score distribution and patient survival status. **(D)** Kaplan–Meier analysis of the OS between the two groups. **(E)** ROC curves to predict the sensitivity and specificity of 1-, 3-, and 5-year survival according to the RMW score.

Supplementary Figure 10 The expression level of 7 RMW score-related gens. **(A–G)** The expression level of ADNP, DYRK1B, GFPT2, MYCNOS, PLCH1, ZFHX4 and ZNF429 in OC cell lines.

Supplementary Figure 11 Stratification analysis of the RMW score in OC. **(A–C)** The proportion of patient age, stage and survival status in high- and low-RMW score groups. **(D–F)** Boxplots for RMW score between different characteristics OC patients, including patient age, stage and survival status. **(G–N)** Kaplan-Meier curves depicted the survival difference between low and high RMW score in the stratified analysis of OC patients.

Supplementary Figure 12 Immune infiltration characteristics between the two RMW score subgroups. **(A)** Heatmap showing the association between RMW score and immune cells. **(B)** The proportion of each immune cell in two RMW score subgroups. **(C)** Correlations between RMW score and immune cell types. **(D)** Heatmap shows a positive and negative correlation between drug targeted genes and RMW score in OC.

Supplementary Figure 13 The Correlation between the RMW score and genetic variations. **(A)** Relationships between RMW score and CSC index (RNAss). **(B)** Relationships between RMW score and CSC index (DNAss). **(C)** TMB in different RMW score groups. **(D)** The waterfall plot of somatic mutation features in high RMW score. **E** The waterfall plot of somatic mutation features in low RMW score.

Supplementary Figure 14 Scatter plots to show the association between different “writers” expression and drug sensitivity.
