# Supplementary material for: Crosstalk of RNA Adenosine Modification-Related Subtypes, Establishment of a Prognostic Model, and Immune Infiltration Characteristics in Ovarian Cancer
Source: Front Immunol. 2022 Jun 28;13:932876. doi: 10.3389/fimmu.2022.932876 (PMC9274011; doi:10.3389/fimmu.2022.932876)
Supplement: Supplementary file 1 [file Table_1.docx]

**Table S1 Summary of 26 RNA Modification writers.**

| **Gene** | **Type** |
| --- | --- |
| ZC3H13 | m6A writers |
| WTAP | m6A writers |
| RBM15B | m6A writers |
| RBM15 | m6A writers |
| METTL3 | m6A writers |
| METTL14 | m6A writers |
| VIRMA | m6A writers |
| TRMT61B | m1A writers |
| TRMT61A | m1A writers |
| TRMT6 | m1A writers |
| TRMT10C | m1A writers |
| CFI | APA writers |
| CLP1 | APA writers |
| CPSF1 | APA writers |
| CPSF2 | APA writers |
| CPSF3 | APA writers |
| CPSF4 | APA writers |
| CSTF1 | APA writers |
| CSTF2 | APA writers |
| CSTF3 | APA writers |
| NUDT21 | APA writers |
| PABPN1 | APA writers |
| PCF11 | APA writers |
| ADARB2 | A-I writers |
| ADARB1 | A-I writers |
| ADAR | A-I writers |

**Table S2 Primer sequence of genes in qRT-PCR**

|  | Forward sequence | Reverse sequence |
| --- | --- | --- |
| ZFHX4 | AAAACGGATGAGCGCAAAAGT | TGGTAAACGATCTCCCCTGTTAG |
| DYRK1B | CACCCCAGGATTCGAGCAAC | TGAGCGAGTCAATTTCGTAGC |
| PLCH1 | AGAGCCAGTAGTACATCATGGT | AACAGATGACAGGTCCAGTTTG |
| GFPT2 | AGACACACTTCGGCATTGC | TTGGCGATGGTCTCTGTATCT |
| ZNF429 | TGCAAGATGAAGCGACATGAA | GCTCTGGGTAAGTGTCAAACAT |
| ADNP | AGGCTGACAGTGTAGAGCAAG | GACTGCCCCATTGAGTGATTTT |
| MYCNOS | GGACACCCTGAGCGATTCAGA | AGGAGGAACGCCGCTTCT |

**Table S3 Prognostic analysis of 10 survival-related DEGs using univariate Cox regression model**

| **id** | **HR** | **HR.95L** | **HR.95H** | **pvalue** |
| --- | --- | --- | --- | --- |
| ACSM3 | 0.7611 | 0.6671 | 0.8683 | 0.0000 |
| ZFHX4 | 1.2416 | 1.1162 | 1.3811 | 0.0001 |
| DYRK1B | 1.8398 | 1.2954 | 2.6131 | 0.0007 |
| PRKG1 | 1.3050 | 1.1391 | 1.4950 | 0.0001 |
| PLCH1 | 0.8128 | 0.7297 | 0.9054 | 0.0002 |
| GFPT2 | 1.2190 | 1.0839 | 1.3708 | 0.0009 |
| ZNF429 | 0.5148 | 0.3773 | 0.7025 | 0.0000 |
| ZFHX4-AS1 | 1.1948 | 1.0871 | 1.3132 | 0.0002 |
| ADNP | 1.9124 | 1.3162 | 2.7788 | 0.0007 |
| MYCNOS | 0.8035 | 0.7087 | 0.9110 | 0.0006 |

**Table S4 47 immune checkpoint blockage-related genes**

**Gene**

IDO1

LAG3

CTLA4

TNFRSF9

ICOS

CD80

PDCD1LG2

TIGIT

CD70

TNFSF9

ICOSLG

KIR3DL1

CD86

PDCD1

LAIR1

TNFRSF8

TNFSF15

TNFRSF14

IDO2

CD276

CD40

TNFRSF4

TNFSF14

HHLA2

CD244

CD274

HAVCR2

CD27

BTLA

LGALS9

TMIGD2

CD28

CD48

TNFRSF25

CD40LG

ADORA2A

VTCN1

CD160

CD44

TNFSF18

TNFRSF18

BTNL2

C10orf54

CD200R1

TNFSF4

CD200

NRP1
