# Supplementary figures and images for: Crosstalk of RNA Adenosine Modification-Related Subtypes, Establishment of a Prognostic Model, and Immune Infiltration Characteristics in Ovarian Cancer
Source: Front Immunol. 2022 Jun 28;13:932876. doi: 10.3389/fimmu.2022.932876 (PMC9274011; doi:10.3389/fimmu.2022.932876)

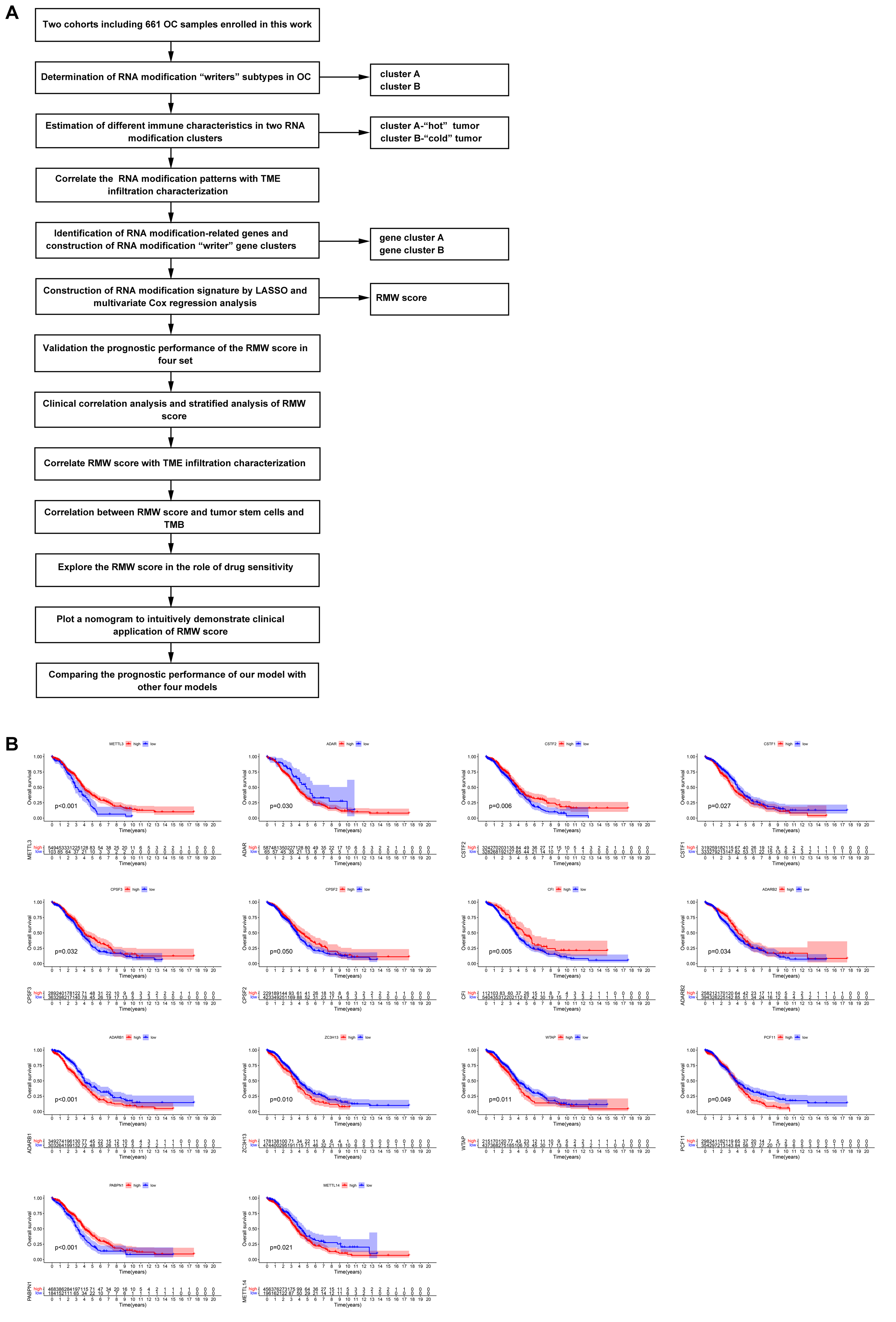

Supplement: Supplementary Figure 1 — Overview of study design and prognostic characteristics of RNA modification writers. (A) Overview of this work. (B) Kaplan-Meier survival analysis of different RNA modification “writers” in OC. [file Image_1.tif]

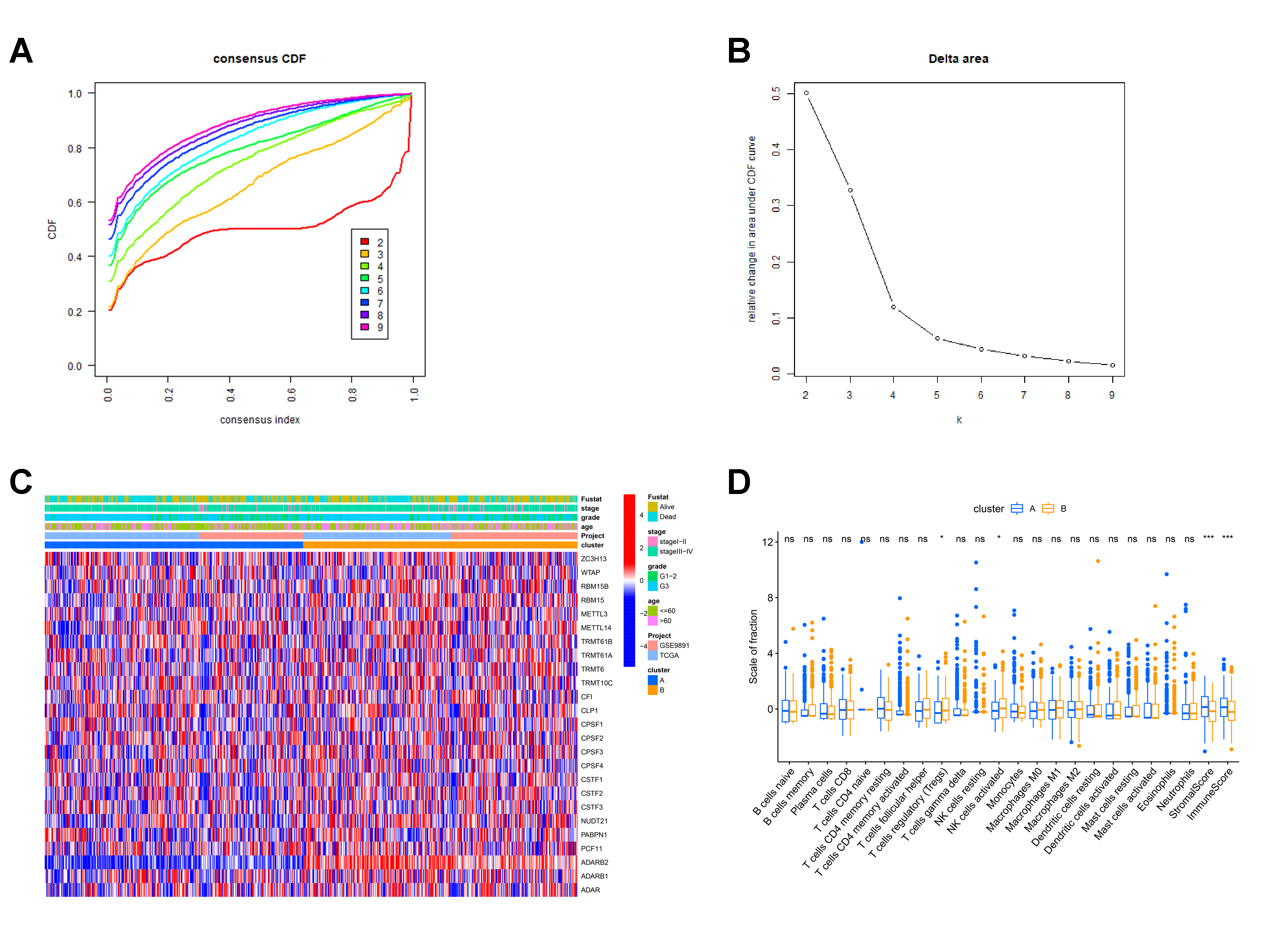

Supplement: Supplementary Figure 2 — Clinical and immune infiltration characteristics in two modification patterns. (A) Cumulative distribution function curves for unsupervised clustering of 26 RNA modification “writers”, k = 2-9. (B) Relative change in area under the CDF curve for unsupervised clustering of 26 RNA modification “writers”, k = 2-9. C Heatmap showing differences in clinicopathologic features and expression levels of RNA modification “writers” between the two modification patterns. (D) The proportion of each immune cell in two modification patterns. [file Image_2.tif]

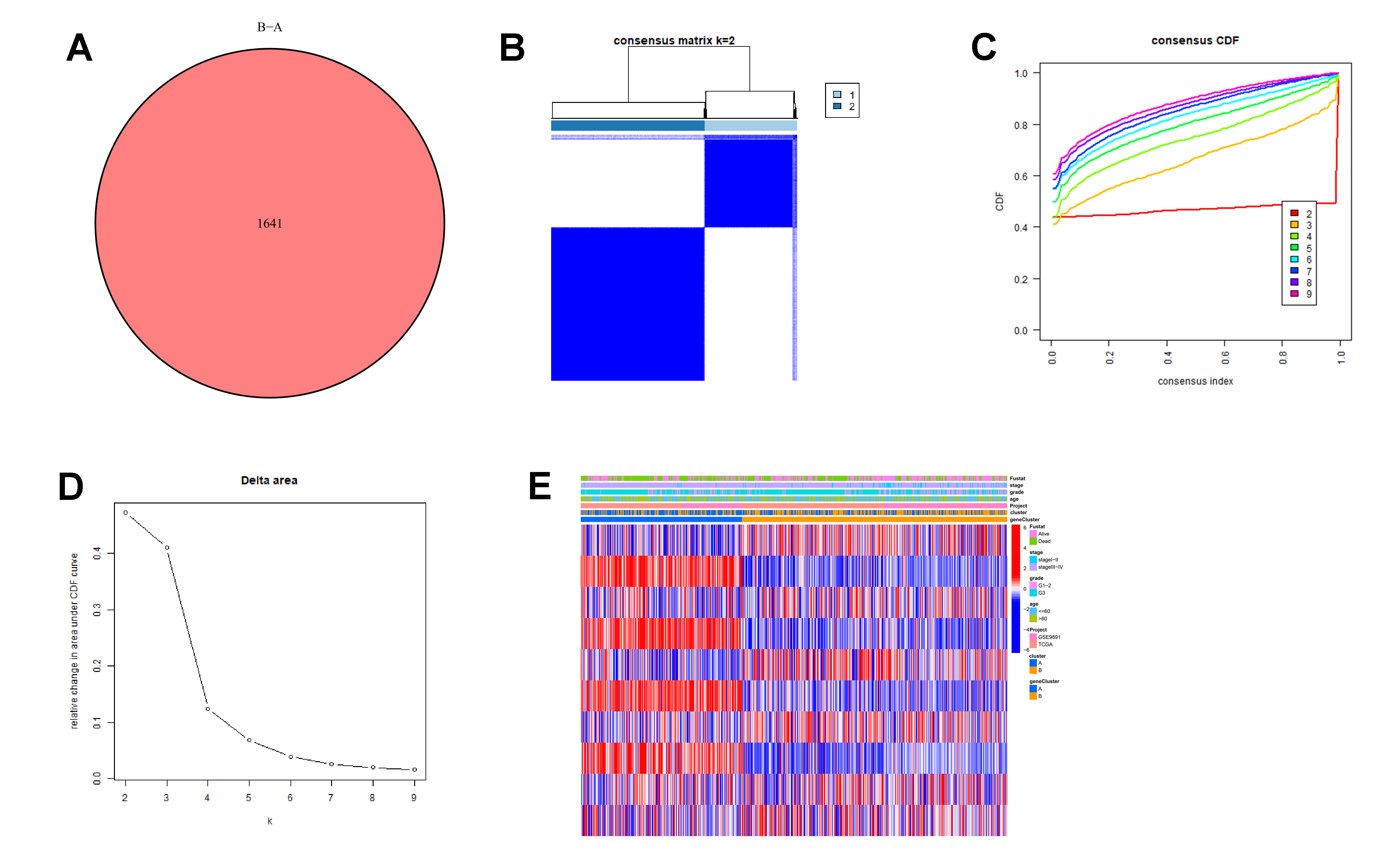

Supplement: Supplementary Figure 3 — Clinical and immune infiltration characteristics in two gene clusters. (A), 1641 RNA modification patterns-related DEGs shown in venn diagram. (B) Heat map of the consensus matrix for the OC sample at k = 2. C Cumulative distribution function curves for unsupervised clustering of DEGs, k = 2-9. (D) Relative change in area under the CDF curve for unsupervised clustering of DEGs, k = 2-9.(E) Heatmap showing differences in clinicopathologic features and expression levels of RNA modification “writers” between gene clusters. [file Image_3.tif]

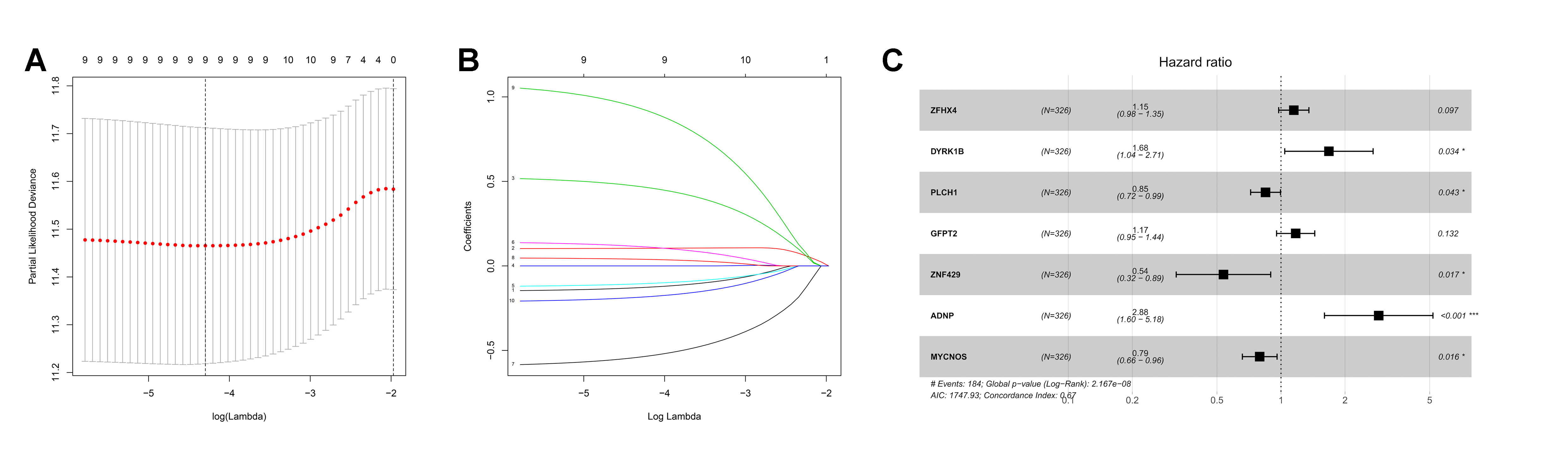

Supplement: Supplementary Figure 4 — Identifying representative candidate prognostic genes. (A-B) The LASSO regression analysis and partial likelihood deviance on the prognostic genes. (C) Forest plot of multivariate cox regression analysis for prognostic genes. [file Image_4.tif]

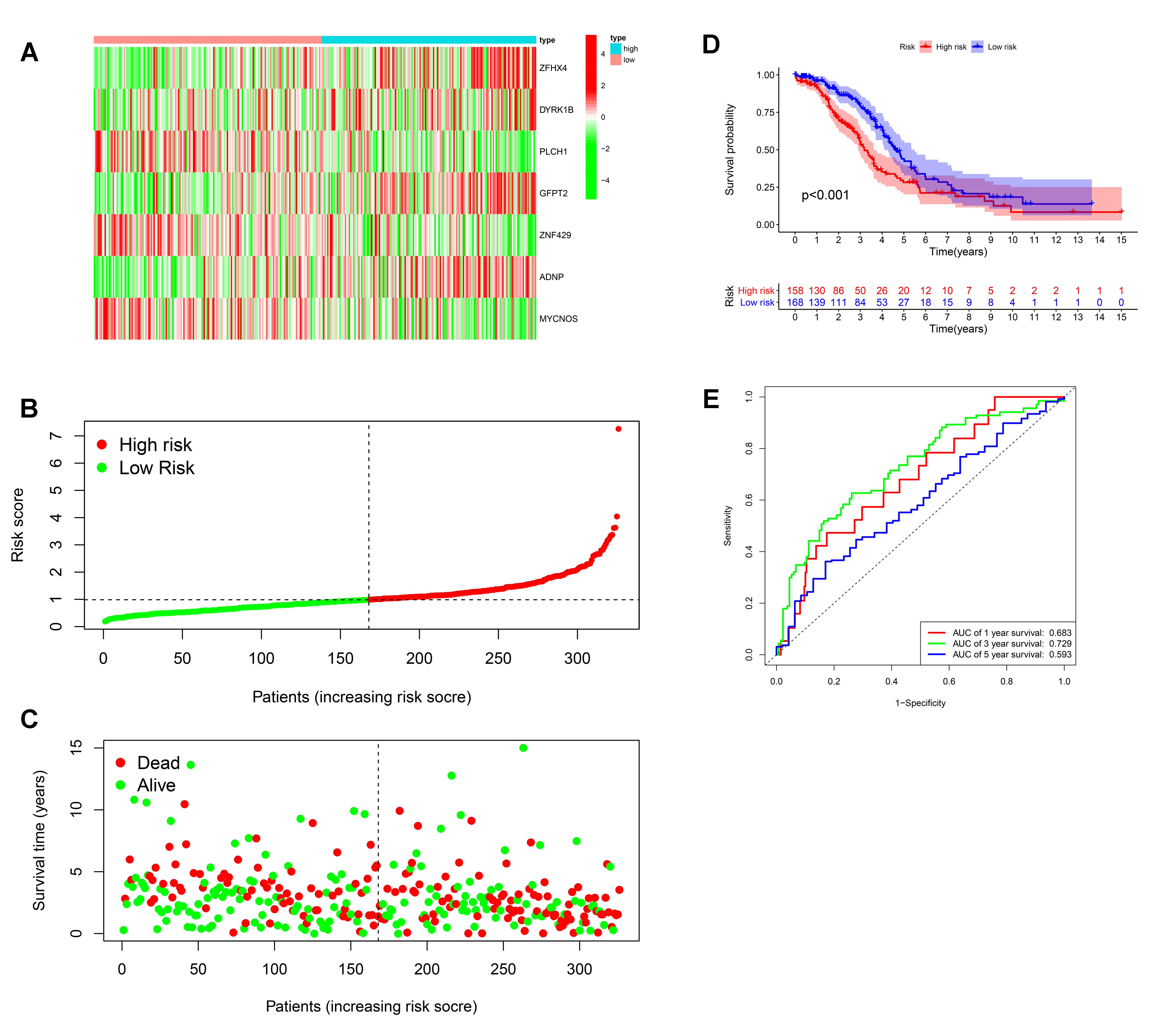

Supplement: Supplementary Figure 5 — Validation of RMW score in testing set. (A) Heatmap shows the distribution of core genes in models between normal and OC tissues. (B) Ranked dot showing the RMW score distribution and patient survival status. (C) Scatter plots showing the RMW score distribution and patient survival status. (D) Kaplan–Meier analysis of the OS between the two groups. (E) ROC curves to predict the sensitivity and specificity of 1-, 3-, and 5-year survival according to the RMW score. [file Image_5.tif]

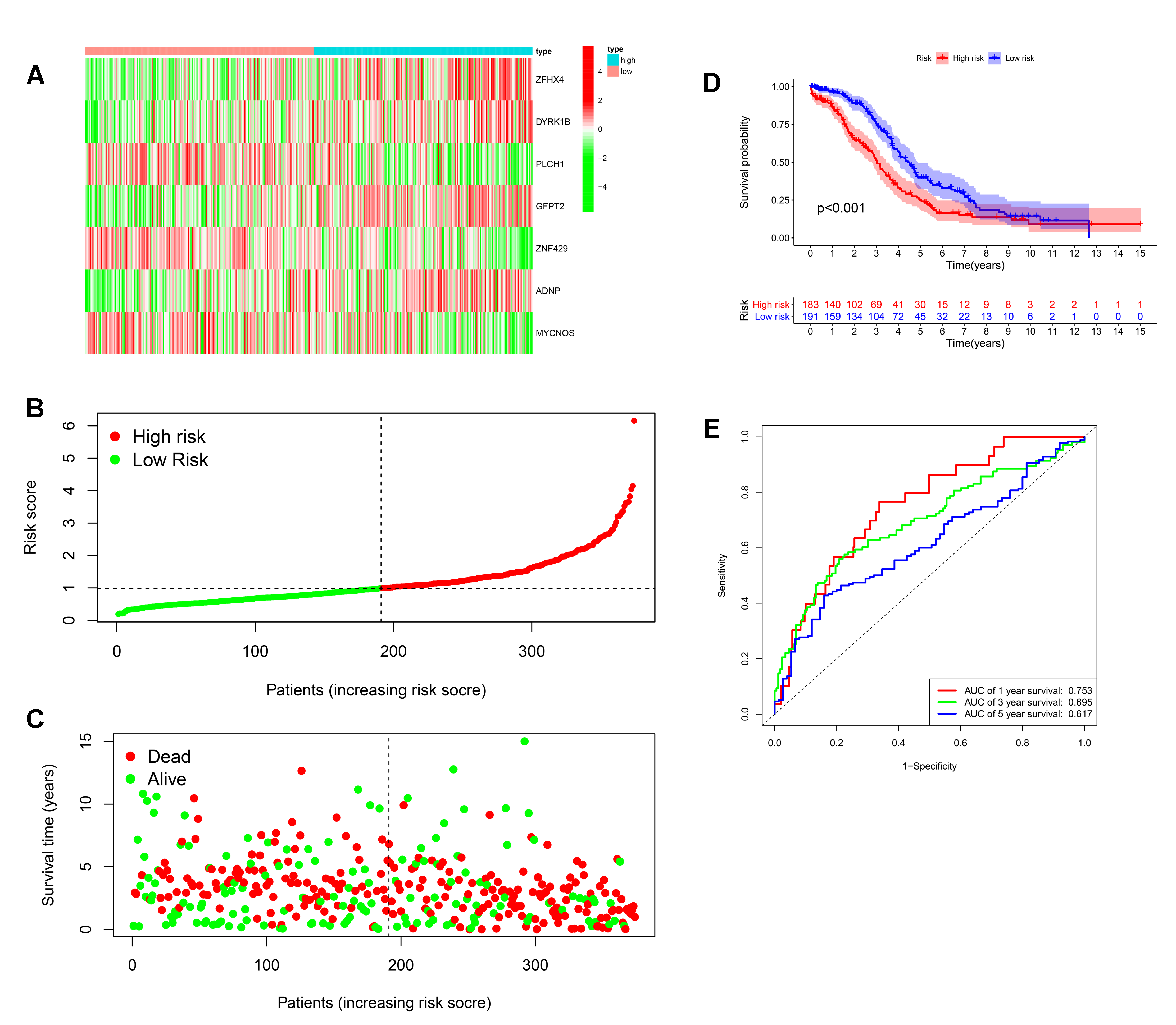

Supplement: Supplementary Figure 6 — Validation of RMW score in TCGA-OC set. (A) Heatmap shows the distribution of core genes in models between normal and OC tissues. (B) Ranked dot showing the RMW score distribution and patient survival status. (C) Scatter plots showing the RMW score distribution and patient survival status. (D) Kaplan–Meier analysis of the OS between the two groups. (E) ROC curves to predict the sensitivity and specificity of 1-, 3-, and 5-year survival according to the RMW score. [file Image_6.tif]

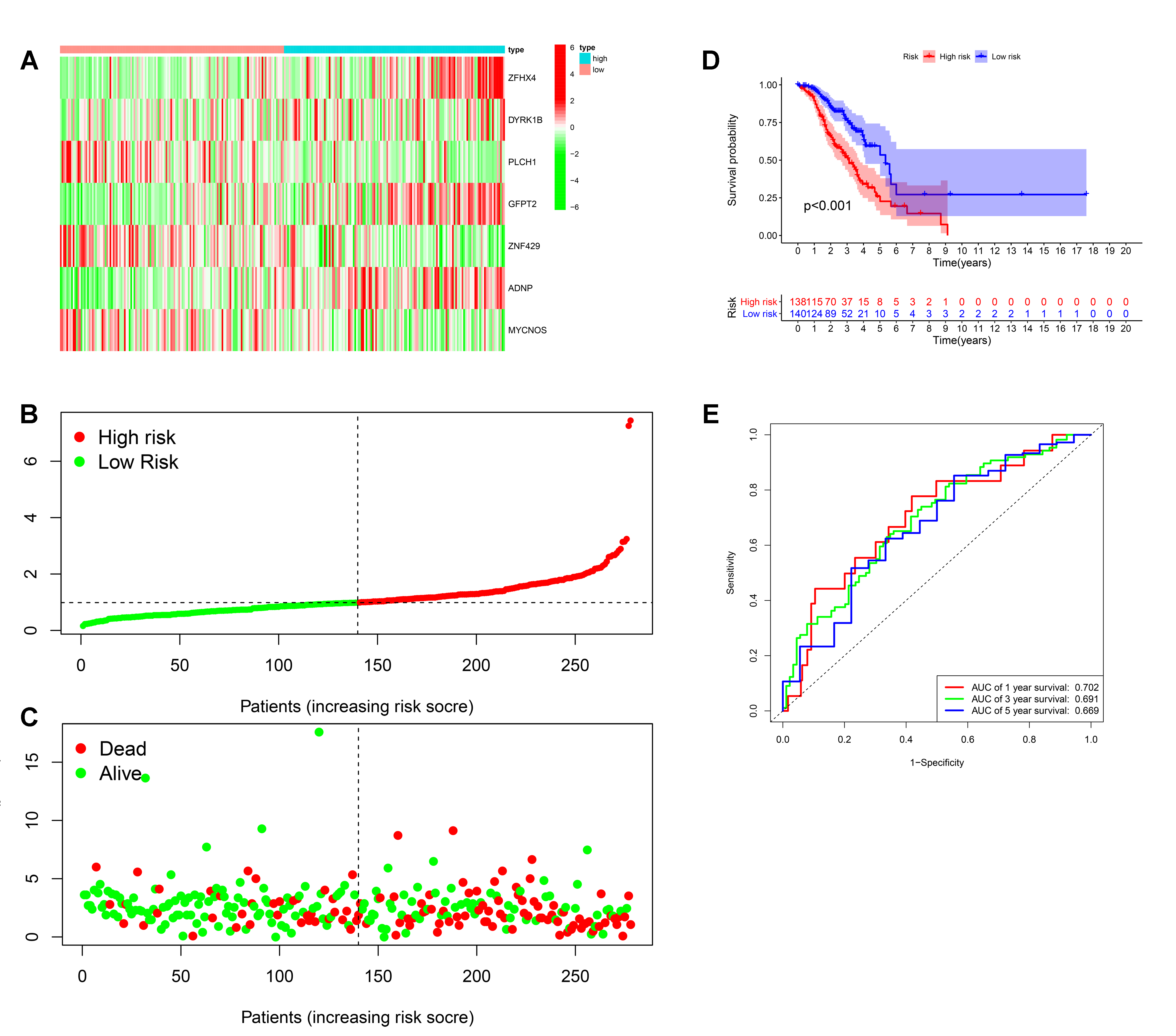

Supplement: Supplementary Figure 7 — Validation of RMW score in GSE9891 set. (A) Heatmap shows the distribution of core genes in models between normal and OC tissues. (B) Ranked dot showing the RMW score distribution and patient survival status. (C) Scatter plots showing the RMW score distribution and patient survival status. (D) Kaplan–Meier analysis of the OS between the two groups. (E) ROC curves to predict the sensitivity and specificity of 1-, 3-, and 5-year survival according to the RMW score. [file Image_7.tif]

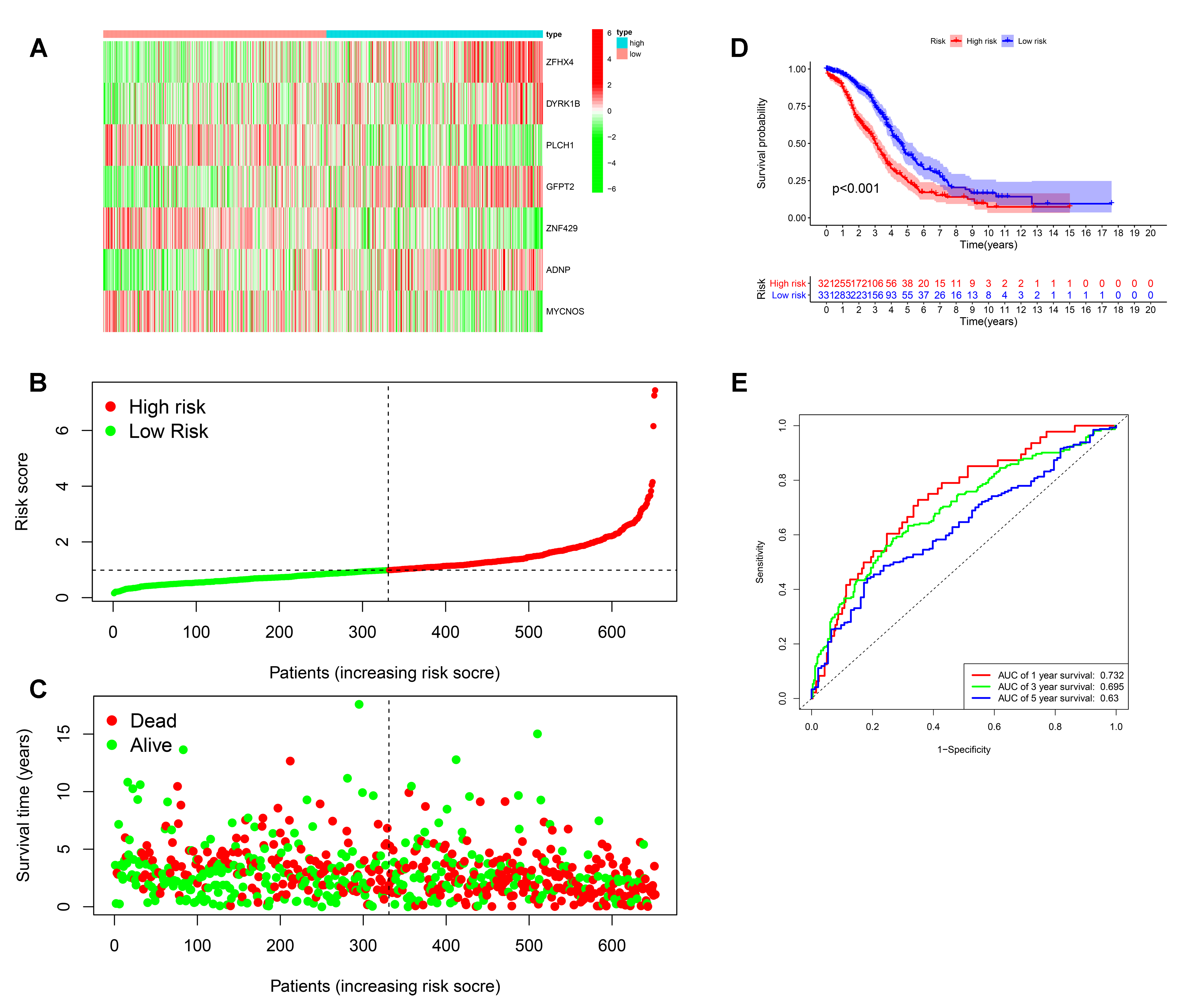

Supplement: Supplementary Figure 8 — Validation of RMW score in whole set. (A) Heatmap shows the distribution of core genes in models between normal and OC tissues. (B) Ranked dot showing the RMW score distribution and patient survival status. (C) Scatter plots showing the RMW score distribution and patient survival status. (D) Kaplan–Meier analysis of the OS between the two groups. (E) ROC curves to predict the sensitivity and specificity of 1-, 3-, and 5-year survival according to the RMW score. [file Image_8.tif]

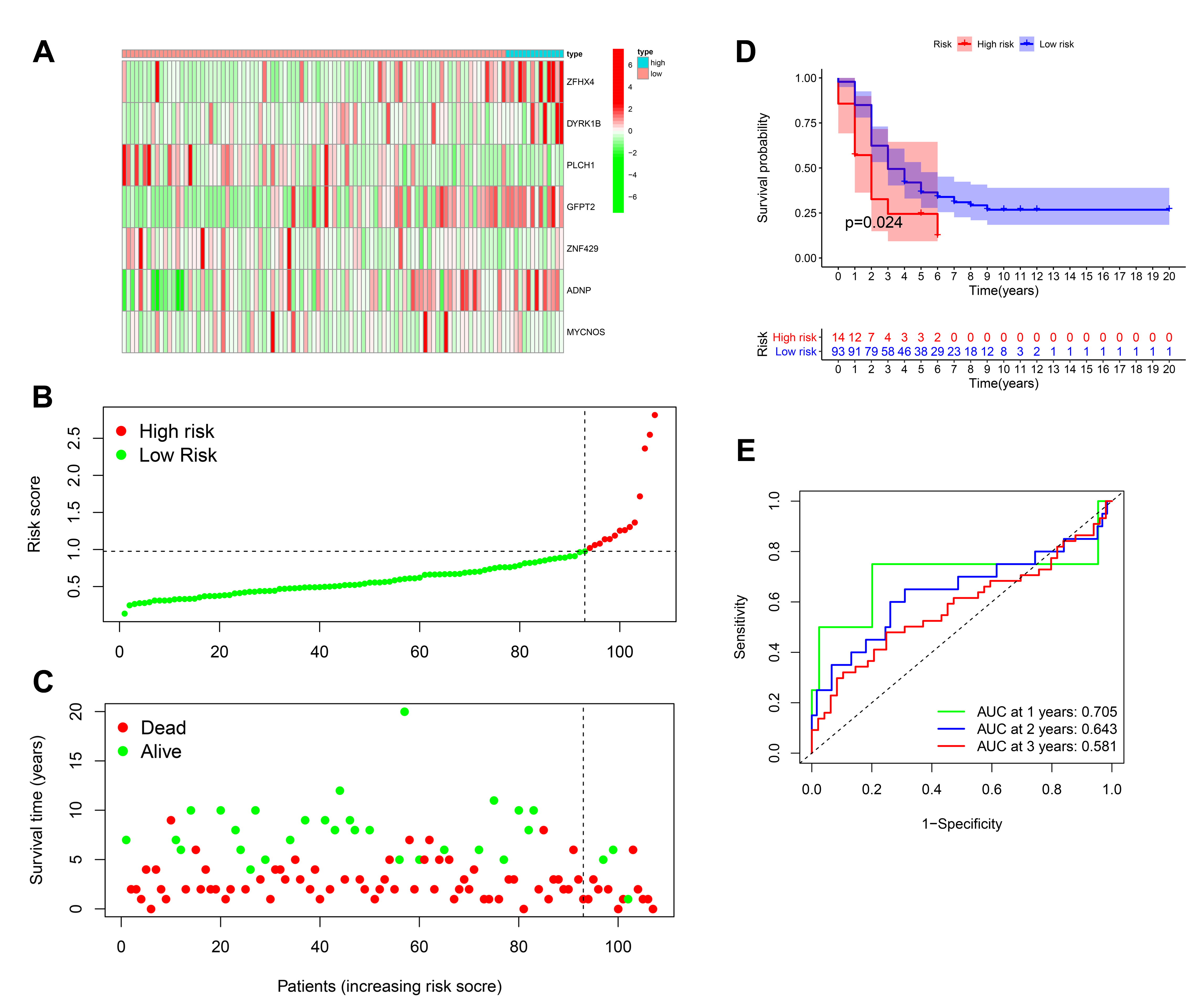

Supplement: Supplementary Figure 9 — Validation of RMW score in GSE26193 set. (A) Heatmap shows the distribution of core genes in models between normal and OC tissues. (B) Ranked dot showing the RMW score distribution and patient survival status. (C) Scatter plots showing the RMW score distribution and patient survival status. (D) Kaplan–Meier analysis of the OS between the two groups. (E) ROC curves to predict the sensitivity and specificity of 1-, 3-, and 5-year survival according to the RMW score. [file Image_9.tif]

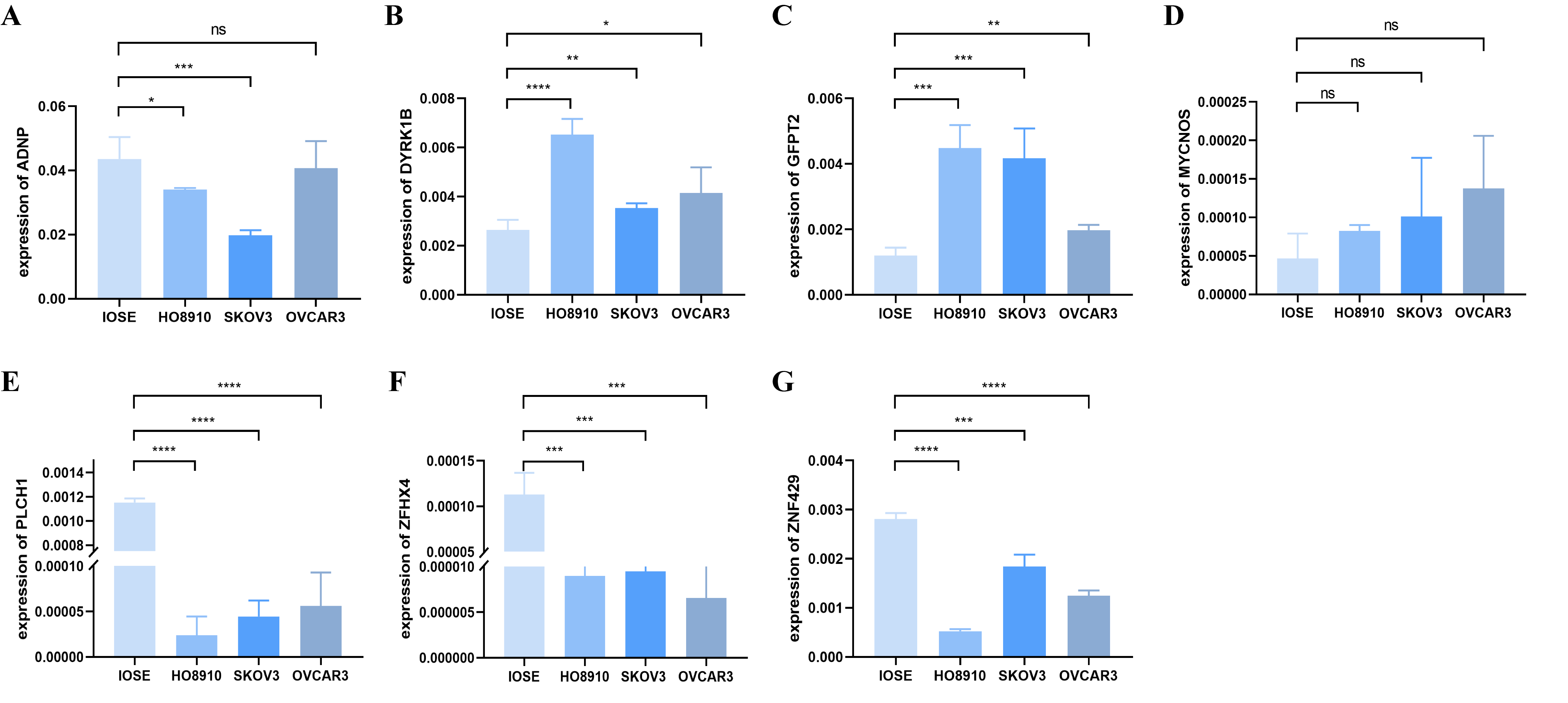

Supplement: Supplementary Figure 10 — The expression level of 7 RMW score-related gens. (A–G) The expression level of ADNP, DYRK1B, GFPT2, MYCNOS, PLCH1, ZFHX4 and ZNF429 in OC cell lines. [file Image_10.tif]

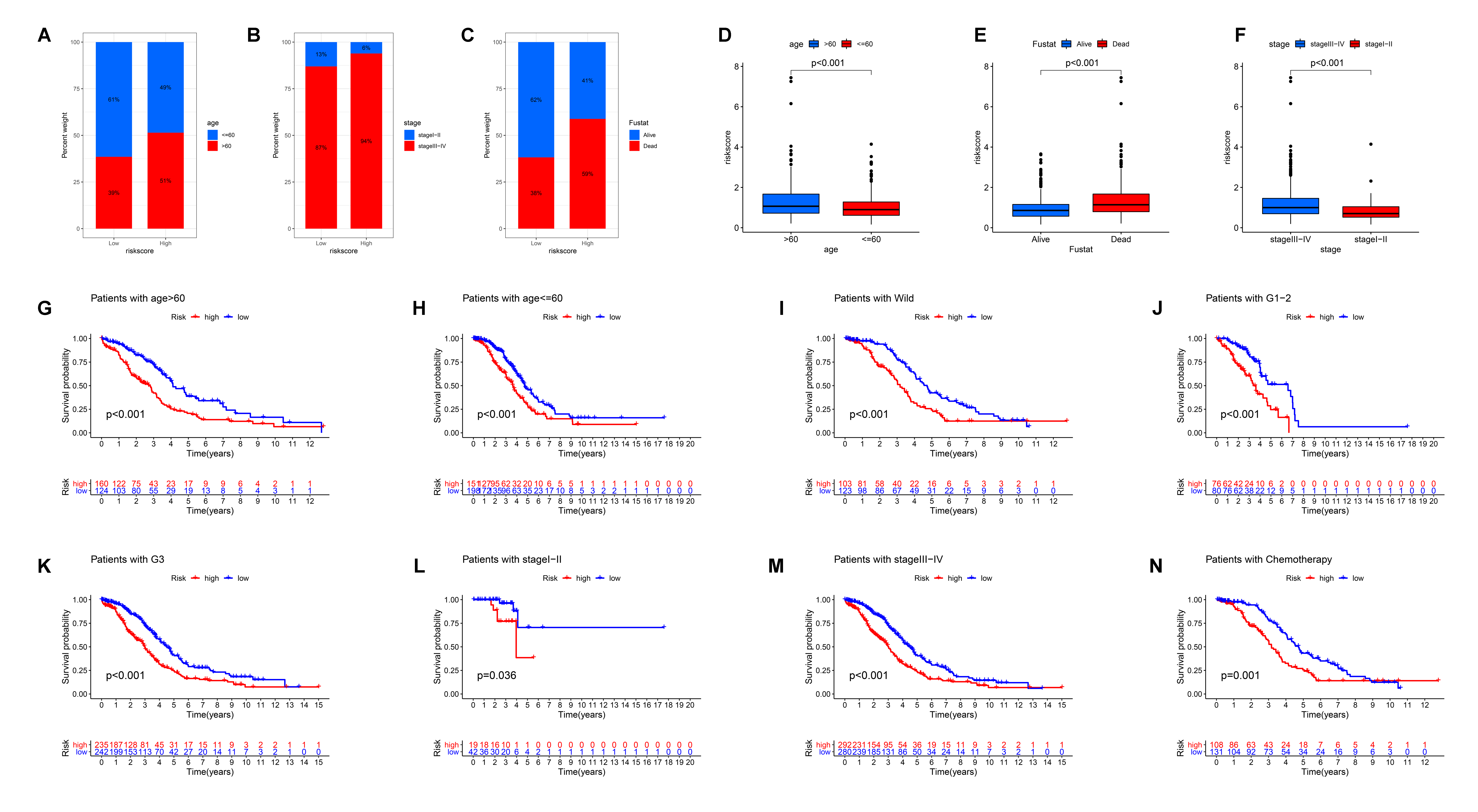

Supplement: Supplementary Figure 11 — Stratification analysis of the RMW score in OC. (A–C) The proportion of patient age, stage and survival status in high- and low-RMW score groups. (D–F) Boxplots for RMW score between different characteristics OC patients, including patient age, stage and survival status. (G–N) Kaplan-Meier curves depicted the survival difference between low and high RMW score in the stratified analysis of OC patients. [file Image_11.tif]

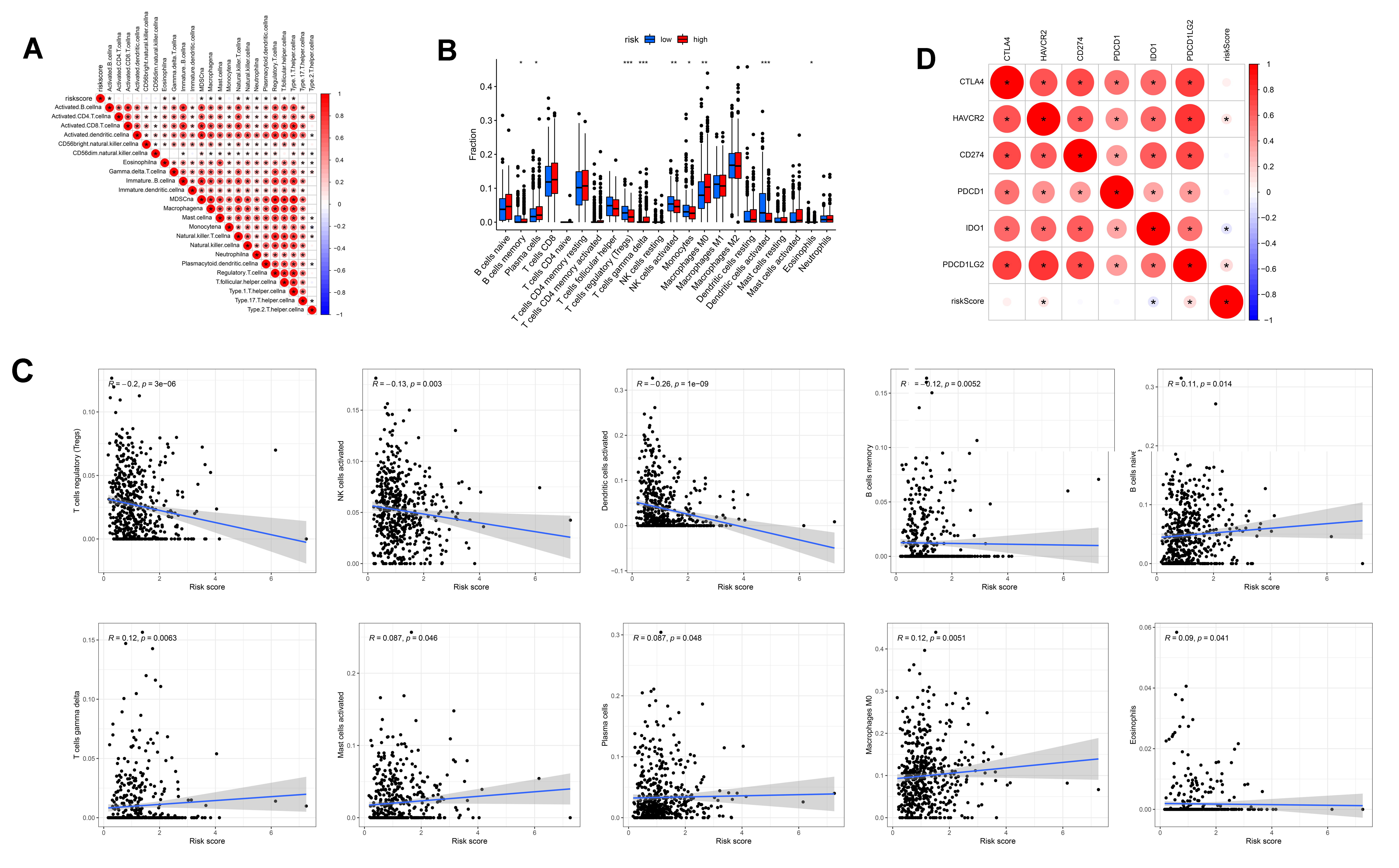

Supplement: Supplementary Figure 12 — Immune infiltration characteristics between the two RMW score subgroups. (A) Heatmap showing the association between RMW score and immune cells. (B) The proportion of each immune cell in two RMW score subgroups. (C) Correlations between RMW score and immune cell types. (D) Heatmap shows a positive and negative correlation between drug targeted genes and RMW score in OC. [file Image_12.tif]

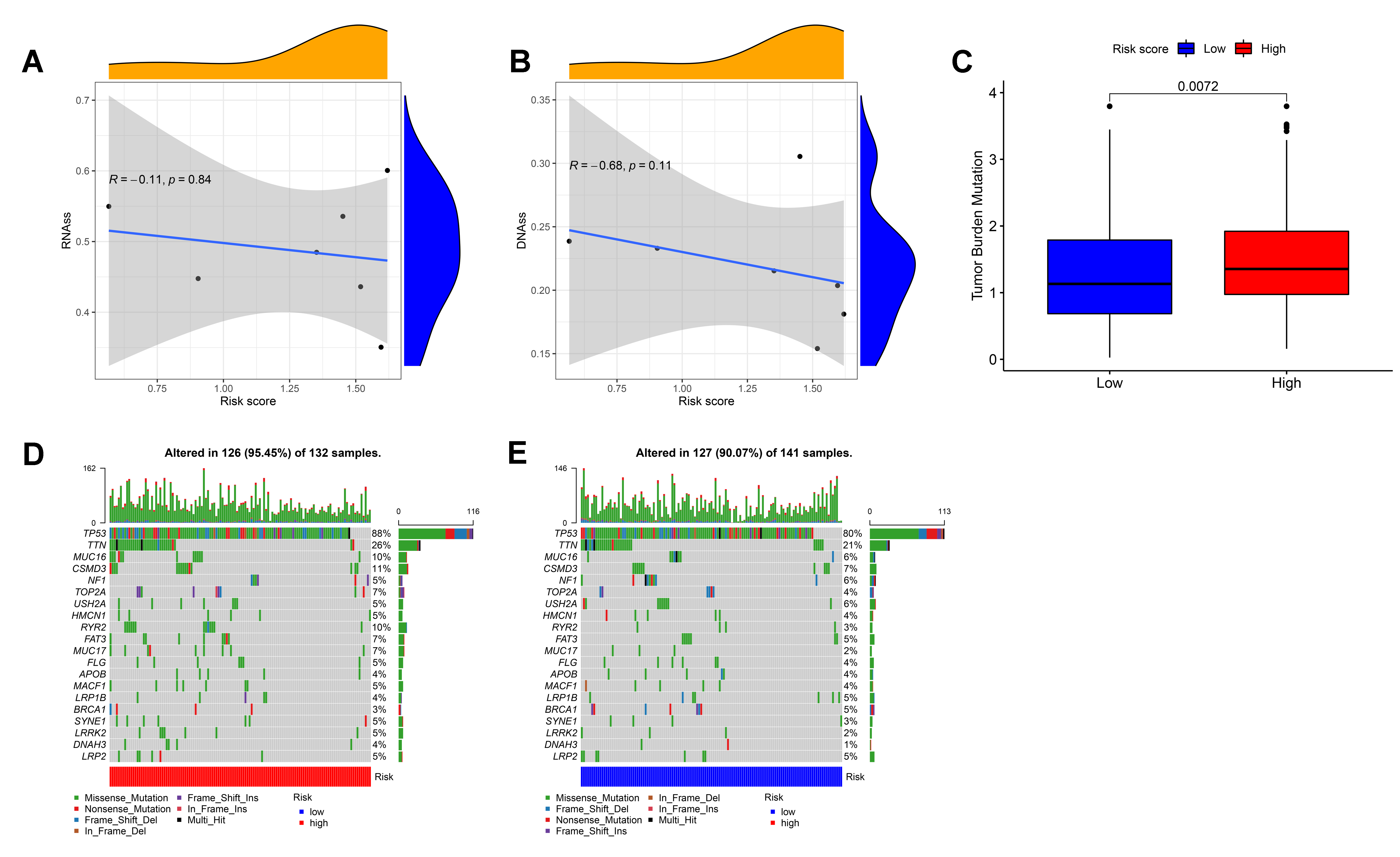

Supplement: Supplementary Figure 13 — The Correlation between the RMW score and genetic variations. (A) Relationships between RMW score and CSC index (RNAss). (B) Relationships between RMW score and CSC index (DNAss). (C) TMB in different RMW score groups. (D) The waterfall plot of somatic mutation features in high RMW score. E The waterfall plot of somatic mutation features in low RMW score. [file Image_13.tif]

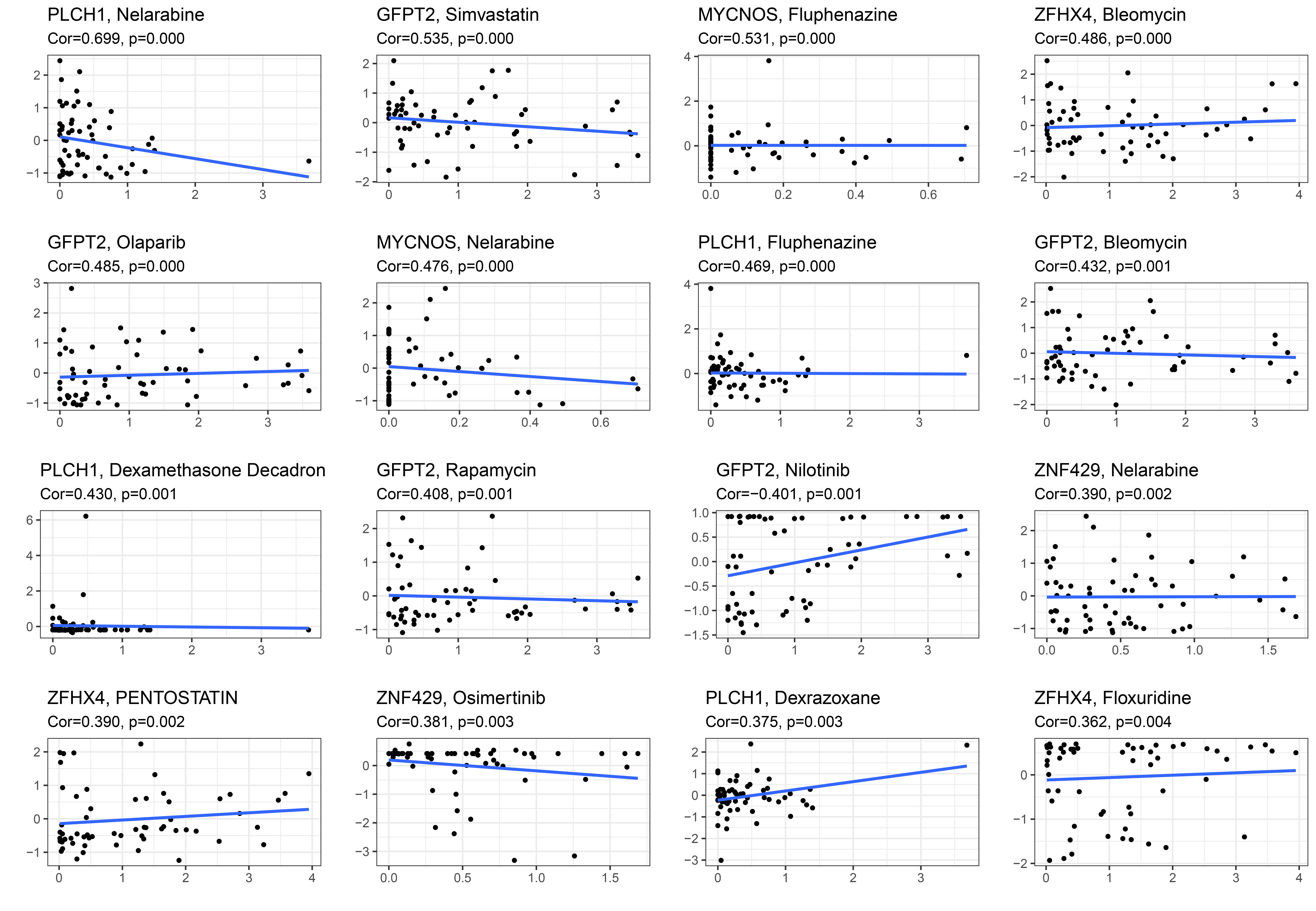

Supplement: Supplementary Figure 14 — Scatter plots to show the association between different “writers” expression and drug sensitivity. [file Image_14.tif]
